# **M**odifying **I**ntestinal Integrity and **M**icro **B**iome in Severe Malnutrition with **Le**gume-Based Feeds (MIMBLE 2.0): protocol for a phase II refined feed and intervention trial

**DOI:** 10.12688/wellcomeopenres.14706.1

**Published:** 2018-08-02

**Authors:** Kevin Walsh, Nuala Calder, Peter Olupot-Olupot, Tonny Ssenyondo, William Okiror, Charles Bernard Okalebo, Rita Muhindo, Ayub Mpoya, Elaine Holmes, Julian Marchesi, Gael Delamare de la Villenaise de Chenevarin, Gary Frost, Kathryn Maitland

**Affiliations:** 1 Department of Paediatrics, Imperial College, London, W2 1PG, UK; 2 Section for Nutrition Research, Department of Medicine, Imperial College, London, W12 ONN, UK; 3 Department of Paediatrics, Mbale Regional Referral Hospital, Mbale, PO Box 1966, Uganda; 4 Mbale Clinical Research Institute, Mbale, PO Box 1966, Uganda; 5 Clinical Trials Facility, KEMRI-Wellcome Trust Research Programme, Kilifi, PO Box 230, Kenya; 6 Division of Computational and Systems Medicine, Imperial College, London, SW7 2AZ, UK; 7 Centre for Digestive and Gut Health, Imperial College, London, W2 1NY, UK; 8 Production and Processing Research Department, Campden BRI Group, Chipping Campden, GL55 6LD, UK

**Keywords:** severe malnutrition, nutritional feeds, African children, gut-barrier dysfunction, legume-based fermentable carbohydrates F75 F100 clinical trial, Uganda

## Abstract

**Background: **Changes in intestinal mucosal integrity and gut microbial balance occur in severe acute malnutrition (SAM), resulting in treatment failure and adverse clinical outcomes (gram-negative sepsis, diarrhoea and high case-fatality). Transient lactose intolerance, due to loss of intestinal brush border lactase, also complicates SAM, thus milk based feeds may not be optimal for nutritional rehabilitation. Since the gut epithelial barrier can be supported by short chain fatty acids, derived from microbiota fermentation by particular fermentable carbohydrates, we postulated that an energy-dense nutritional feed comprising of legume-based fermentable carbohydrates, incorporated with lactose-free versions of standard World Health Organization (WHO) F75/F100 nutritional feeds will enhance epithelial barrier function in malnourished children, reduce and promote resolution of diarrhoea and improve overall outcome.

**Methods:** We will investigate in an open-label trial in 160 Ugandan children with SAM, defined by mid-upper arm circumference <11.5cm and/or presence of kwashiorkor. Children will be randomised to a lactose-free, chickpea-enriched feed containing 2 kcal/ml, provided in quantities to match usual energy provision (experimental) or WHO standard treatment F75 (0.75 kcal/ml) and F100 (1 kcal/ml) feeds on a 1:1 basis, conducted at Mbale Regional Referral Hospital nutritional rehabilitation unit. The primary outcomes are change in MUAC at day 90 and survival to day 90. Secondary outcomes include: i) moderate to good weight gain (>5 g/kg/day), ii) *de novo* development of diarrhoea (>3 loose stools/day), iii) time to diarrhoea resolution (if >3 loose stools/day), and iv) time to oedema resolution (if kwashiorkor) and change in intestinal biomarkers (faecal calprotectin).

**Discussion:** We hypothesize that, if introduced early in the management of malnutrition, such lactose-free, fermentable carbohydrate-based feeds, could safely and cheaply improve global outcome by reducing lactose intolerance-related diarrhoea, improving mucosal integrity and enhancing immunity, and limiting the risk of systemic infection and associated broad-spectrum antibiotic resistance.

**Registration:** ISRCTN 10309022.

## Introduction

Severe acute malnutrition (SAM) accounts for 1.5 million deaths worldwide annually ^1^. Undernutrition, highlighted in a Lancet series ^2^, is a greatly neglected area of research despite contributing to around 60% of childhood deaths. Community-based care with ready-to-use therapeutic food for children with *uncomplicated* malnutrition has markedly improved recovery rates; but little progress has been made for children hospitalised with SAM in whom mortality remains high ^3^. Recommended nutritional support has shown improvements in growth but these often do not translate to improving mortality and relapse rates, indicating that current strategies are not working in practice. In order to understand why current nutritional feeds have limited impact on longer-term mortality rates despite nutritional recovery, we need a new approach to understanding the complex interaction between nutrition, the gut and its associated microbiome. For example, in African children diarrhoea complicates 65% of these children and is associated with high morbidity and mortality rates (20%) ^3, 4^. SAM causes disruption of normal intestinal flora ^5^, increased turnover of vital nutrients, disruption of gut barrier function, impaired mucosal immunity and increased risk of gram negative bacteraemia ^4^, which together form a vicious cycle ^6^.

### Lactose intolerance in SAM

The current recommended formulae by WHO, initially F75 followed by F100, are milk-based feeds and their major carbohydrate source are disaccharides (a mixture of maltodextrin, sucrose and lactose), which can cause osmotic diarrhoea ^7^ since they normally are hydrolysed by disaccharidases localized at the tip of small intestinal villi. Both lactose intolerance ^8, 9^ and intestinal atrophy have been demonstrated in children with SAM ^10– 12^. Removing lactose and replacing it with better-tolerated simple carbohydrates that do not rely on brush border enzymatic activity for digestion and absorption may be highly beneficial in preventing and ameliorating diarrhoea in SAM.

### Benefits of non-digestible fermentable carbohydrates

Numerous studies have shown that intestinal mucosal integrity and gut microbial balance can be restored by inducing fermentation in the gastrointestinal tract ^13^. Fermentable carbohydrates are increasingly being investigated as potential adjuncts to improve the balance of normal gut flora and positively influence the immunological and metabolic function of the gut ^14^. Non-digestible fermentable oligosaccharides induce favourable colonic microbiota fermentation ^15^, leading to the generation of short-chain fatty acids (SCFA), which have a positive influence on gut integrity and nutritional health by improving energy yield, production of vitamins and the stimulation of gut homeostasis, including anti-pathogen activities ^16, 17^. Preliminary data collected from children with SAM by a multi-disciplinary team at Imperial College (SMIP study) involving specialists in international child health (K.M.), nutrition (G.F.), metabonomics (E.H.) and colonic bacterial metataxonomics (J.M.) suggest that there are changes in the inter-relationships of normal gut microbes, intestinal permeability, and markers of gut barrier dysfunction and immune dysregulation in SAM which effect clinical outcome (manuscript in preparation) and are therefore potential targets for intervention. 

**Figure 1.**
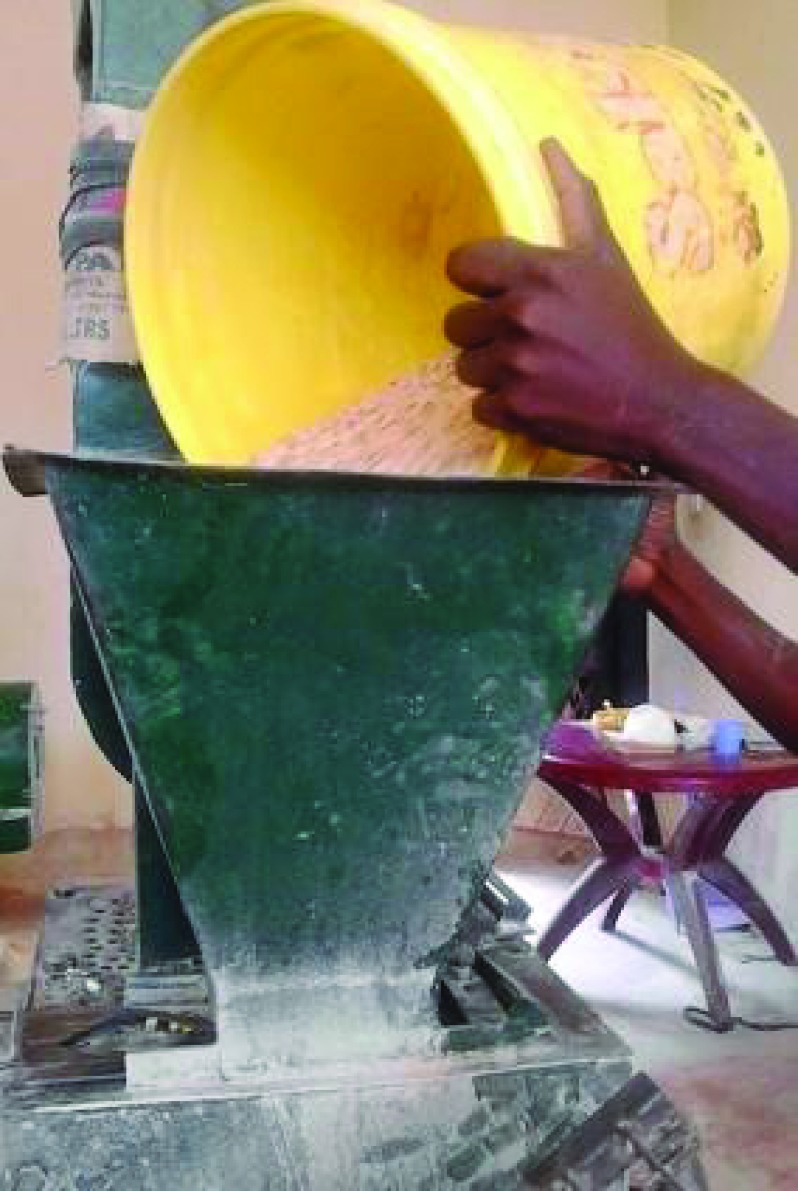
“Posho” mill grinding legumes to create flour.

In the MIMBLe (Modifying Intestinal Integrity and Micro biome in Severe Malnutrition with Legume-Based Feeds) pilot study we tested a locally made cowpea flour using a “Posho” mill, which was added to standard nutritional feeds (as in Figure 1) (Pan African Clinical Trial Registry: PACTR201805003381361). This small study demonstrated that the feed is well tolerated when added to the nutritional recovery programme, and increased bacterial diversity compared to standard feeds. We also demonstrated decreases in anorectic gut hormones (manuscript in preparation).

### Sources of non-digestible fermentable carbohydrate

Legumes, which contain a mixture of different types of non-digestible fermentable carbohydrates, have been demonstrated to augment microbial communities that enhance SCFA production ^18^. Furthermore, these legumes form part of the traditional East African diet consumed by children ^19^ and a number of current research programmes in East Africa are expanding legume growth to improve the environmental impact of agriculture (nitrogen fixing) ^20^, meaning that this treatment will be both acceptable and readily available to local communities. One potential commonly consumed food with prebiotic effect is chickpea. Chickpea-based follow-on formulae have been explored as a potential prevention for undernutrition ^21^.

### Summary and importance

There are multiple reasons why nutritional support, at an early stage in the management of SAM, tailored towards prevention of diarrhoea and restoring gut mucosal integrity and manipulation of the gut microbiome could enhance resilience to secondary infection and morbidity during re-feeding. Legumes, through the support of microbiota networks to produce short chain fatty acids, offer a safe therapeutic adjuvant that is easily and cheaply sourced in East Africa, and other regions with a high burden of severe malnutrition. Additional removal of lactose from therapeutic feeds should reduce incidence of osmotic diarrhoea, and related morbidity.

The planned research will bring new knowledge to the field of severe malnutrition, thereby addressing a major treatment gap, which is a *high priority research issue and a significant barrier to progress in the reduction of mortality.* The study has the potential to suggest new therapies for enhancing recovery of gut mucosal function and resilience to infection through the modulation of the gut microbiome, thereby reducing mortality and morbidity in SAM. As the interventional feed from the original MIMBLE pilot is being refined in conjunction with a specialist food company (Campden BRI), this offers an opportunity to improve the food safety profile, product utility, and further optimise the feed by removing lactose.

## Study protocol

### Justification for the study

This phase II study will be the first step in evaluating whether giving children with severe acute malnutrition an altered nutritional feed helps repair the gut and encourage the healthy microbiome, which may reduce illness or improve recovery. In the experimental arm (legume-based feed) the standard nutritional feed that all children with severe malnutrition receive routinely will be altered by removal of lactose and adding a chickpea flour. Chickpea flour contains fibre/roughage that can help encourage SCFA production leading to modulation of the gut microbiome, thereby reducing mortality and morbidity in SAM.

### Hypothesis

We hypothesize that legume-enriched, lactose-free nutrition support will improve outcomes in children with severe acute malnutrition, compared with standard nutritional rehabilitation milks as recommended by the WHO.

### Objectives

To generate additional proof supporting the hypothesis that non-digestible fermentable carbohydrate in the form of chickpea flour will have positive effects on intestinal mucosal integrity and permeability, gut inflammation, gut microbiome and clinical outcomes in children with severe acute malnutrition compared to children receiving standard nutritional feeds. Also to provide evidence that a lactose-free feed will reduce the incidence and encourage the resolution of diarrhoea, which is a major complicating factor in SAM.

### Design and methodology

The study will be conducted in Mbale Regional Referral Hospital (MRRH), Uganda. MIMBLE 2 progress at the time of writing is detailed in the ‘Study progress’ section below.

### Study design

A single-centre, open-label pilot phase II trial comparing a lactose-free, chickpea enriched feed containing 2 kcal/ml, provided in quantities to match usual energy provision (experimental) to WHO standard treatment F75 (0.75kcal/ml) and F100 (1kcal/ml) feeds randomised on a 1:1 basis.

### Study location

The study will be conducted at the Paediatric Nutrition Unit at Mbale Regional Referral Hospital, Eastern Uganda. The 17-bedded nutrition unit in Mbale admits 300 children each year with severe acute malnutrition. Most of the clinical services provided by the paediatric department are free of charge to the patients in accordance with the government policy of no cost sharing in public hospitals.

### Study population

160 children aged > 6 months to 5 years. *Inclusion criteria:* signs of severe malnutrition one of (i) Marasmus (defined by mid-upper arm circumference (MUAC) <11.5cm) and/or Kwashiorkor (defined as symmetrical pitting oedema involving at least the feet irrespective of WHZ score or MUAC) and a guardian or parent willing/able to provide consent. *Exclusion criteria:* children with severe acute malnutrition with a very high risk of death due to a comorbidity e.g. malignant disease or terminal illness will be excluded.

### Sample size

Mid-upper arm circumference (MUAC) has been selected as the primary criterion for nutritional recovery because it predicts mortality better and is less affected by oedema than other anthropometric measures, and is also a good index of muscle mass. A recent trial of antimicrobial prophylaxis in Kenyan children admitted with severe malnutrition baseline mean MUAC was 10.6 cm (SD 1.06) and at 90 days, 12.2 cm (SD 1.35), a mean change of 1.6 cm (SD 1.1) nutritional recovery at 90 days ^22^. The overall sample size will be 160 children which will include 80 in each study arm. From the original MIMBLE trial, safety in the study arms were comparable to the standard intervention (manuscript in preparation). As a Phase II study, this study is designed to provide adequate proof of principal that the modified nutritional feed provides clinical, physiological and biological evidence of benefit to the child in terms of nutritional rehabilitation and the predicted effect on biological markers of gut-barrier dysfunction and on the intestinal microbiota. Additionally, it will compare the safety and tolerability of the modified nutritional feed against the current standard of care. The data will thus inform the design and effect size for future pragmatic trials. Formal sample sizes were not calculated; since the study was designed to provide safety data and some indication of the likely efficacy of the modified feed compared to standard of care. We thus aimed to study 160 children in total, which is realistic given the timeframe and funding available for the study. 

### Study methods and procedures

***Screening procedure.*** Dedicated trial research nurses will be employed to conduct the study. Eligible children will be identified by the nurse and clinician on duty and registered in the eligibility screening log. A member of the trial team will then perform a structured clinical assessment of WHO anthropometric and clinical criteria to confirm severe acute malnutrition and any exclusion criteria.

Details of those fulfilling the entry criteria will be entered onto a screening form, while reasons for non-eligibility will be added to the eligibility screening log. It is anticipated that this process will take approximately 10 minutes.

***Consent.*** Prospective written, informed consent will be sought from parents or guardians of children. Parents or guardians will be given an information sheet in their usual language containing details of this pilot study. The sheet will be read aloud to those who are unable to read. Parents and guardians will be encouraged to ask questions about the trial prior to signing the consent form ( Supplementary File 1). For parents who are unable to sign the consent form, a thumbprint will be required and a witness will be required to sign the consent form. Supplementary File 2 contains the information given to the parents/guardians of each child in the study.

***Randomisation.*** Randomisation is in permuted blocks. The sequence was generated by a data manager at KEMRI Wellcome Trust Research Programme (KWTRP), Kilifi, Kenya. This sequence was used, by a study administrator, to prepare cards with treatment allocation (standard or altered standard with chickpea flour) at the Clinical Trial Facility, KWTRP, Kilifi, Kenya. The randomisation cards were sent to MRCI, Mbale. The cards will be kept in numbered, sealed opaque envelopes, each signed across the seal. The randomisation cards are numbered consecutively and the dedicated MIMBLE clinical trial team will select the next card in the sequence ie cards will be opened in numerical order when enrolling a patient in the clinical trial.

***Study interventions.*** Children will be randomly allocated on a 1:1 basis to:

- Control arm: World Health Organization recommended F75 (0.75 kcal/ml) and F100 (1 kcal/ml) feeds
- Experimental arm: Lactose-free, chickpea enriched feed containing 2 kcal/ml, provided in quantities to match energy provision in standard treatment.

For the control arm, children will receive the standard inpatient hospital management as per national guidelines. Briefly, children will initially receive F75 therapeutic milk at a rate of 130 ml/kg/day, provided at 4-hour intervals. When the child has stabilized (resolved hypoglycaemia, resolved hypothermia and demonstrating appetite) they will be transitioned onto F100 therapeutic milk at the same rate. The rate may be increased by 10 ml per feed, until a maximum rate of 200 ml/kg/day has been achieved. If the child is oedematous, F75 will be commenced at 100 ml/kg/day with 4-hourly feeds until stabilized (the above criteria and the resolution of oedema). When stable the child will transition to F100 formula at the same rate, and if tolerated increase to 130 ml/kg/day. If there is no indication of fluid overload, the feed may be increased by 10 ml per feed to a maximum of 200 ml/kg/day.

For the experimental arm, children will receive pre-packaged modified feeds which are lactose-free and include chickpea (gram) flour as a source of fermentable carbohydrate. The feeds contain: lactose-free skimmed milk powder (7.25%), rapeseed oil (11.5%), gram flour (10%), sugar (9%) and water. All ingredients were sourced from established EU suppliers and passed all required safety tests for human consumption as appropriate for each ingredient (contaminants, pesticides, toxins, bacterial contamination etc.). The final feed contains (per 100 ml): 200 kcal, 18 g total carbohydrate, 5.6 g protein, 12g fat, and 0.4 g resistant starch. The quantity of feed provided will match the total amounts of energy and protein that would be received in the control arm, and additional water provided to match the fluid received.

Treatment with control/experimental treatment will be for 14 days duration, followed by standard treatment outpatient as required. This includes provision of ready to use therapeutic feeds until child has recovered, defined by a weight-for-height z-score >−2. The duration of this is variable.

### Clinical management and monitoring

***Clinical monitoring.*** Clinical monitoring will be in line with standard care at a minimum of twice daily temperature and pulse oximetry.

***Anthropometric indices.*** Children will have admission their height or length measured (using a standard height meter or a dedicated length board) and daily weight and MUAC measurements until discharge performed at the same time each day. These measurements will be repeated on day 28 and day 90 ( Table 1).

**Table 1.** Assessment & investigation flow chart.

Time	Adm	Day 1	Day 7	Day 28	Day 90	Discharge
Consent + patient information leaflet	X					
Anthropometry (weight, MUAC)	Daily as inpatient			X	X	X
Height	X			X	X	X
Clinical observations	Daily as inpatient			X	X	X
Fluid balance	Daily as inpatient					X
Stool Assessment	Daily as inpatient			X	X	X
24-Dietary recall	X			X	X	
Length of stay						X
**Laboratory investigations**						
Haematology	X	X	X	X	X	
Biochemistry	X	X	X	X	X	
Glucose	X	X				
Lactate	X					
Malaria test	X					
HIV testing	X					
Urine dipstick (Multi-stick)	X					
Stool microscopy	X					
**Stored samples**						
**For Metabolomics and SCFA (NMR, GC-MS)**						
Plasma		X	X	X	X	
Urine		X	X	X	X	
Stool		X	X	X	X	
**For Metataxonomics (16s rRNA)**						
Stool		X	X	X	X	
**For Intestinal Cell Injury (Faecal Calprotectin)**						
Stool		X	X	X	X	

***Stool assessment.*** Frequency and consistency of stool will be assessed daily until discharge and at day 28 and day 90.

***Dietary recall.*** Children and/or parents/guardians will be asked to complete a 24-hour multi-pass dietary recall via a structured interview to assess the nutritional intake of children on admission, day 28 and day 90 as most children will have progressed onto normal diet with additional RUTF. Intake of standard and modified feeds will be recorded daily during the inpatient episode.

***Day 28 and day 90 follow-up.*** Children will be invited to return to the hospital for a follow-up visit on day 28 and day 90 as a day case which will last approximately 2 hours. This will include a clinical review, anthropometric measurements, and the study investigations outlined above.

***Laboratory investigations.*** Following consent and randomisation, routine admission blood samples will be taken, in accordance to routine clinical practice, for the following investigations: full blood count, clinical chemistry, lactate, glucose, blood culture, malaria status and HIV status. As per WHO guidance all parents will be approached to HIV testing. HIV testing will be conducted with provision of verbal parental or guardian consent. Pre- and post-test counselling will be done in accordance with routine practice. The total admission blood sampling volume equates to 10 ml, the majority of which is for clinical purposes and only includes an extra 3 ml for plasma storage. Routine bloods will also be taken on day 7 and day 28 and day 90 which again will include an extra 3 ml for plasma storage. Plasma will be used for short chain fatty acid and metabolite analysis ( Table 1).

At admission, day 7, day 28 and day 90 stool and urine samples will be taken and be stored. The total volume of urine and stool collected across the study will be 30 ml and 30 g, respectively. Stored samples will be used for metabolite analysis (urine and stool), faecal calprotectin (stool), reducing substances (stool) and DNA extraction for 16S rRNA metataxonomics (stool).

 All stored samples will be sent to Imperial College, London to assess the following:

- Intestinal cell injury, by faecal calprotectin assays.
- Changes in gut microbiota, by 16S rRNA analysis for species related to gut barrier function e.g. *Bifidobacterium* and *Lactobacillus*.
- Examination of changes in host and bacterial metabolic products, by nuclear magnetic resonance spectroscopy and gas chromatography–mass spectrometry.

### Standard case management: in hospital

All study patients will be otherwise managed and clinically monitored as per national guidelines; World Health Organization Guideline- Updates on the management of severe acute malnutrition in infants and children (2013). This includes management of dehydration, hypothermia, hypoglycaemia, dermatosis, treatment of infection, eye care, anaemia and vitamin and mineral supplementation. The criteria to transition from F75 to F100 will be as per national guidelines. The discharge criteria from hospital will also be as per national guidelines.

### Co-primary outcome measures

1. Change in MUAC at Day 90
2. Survival to Day 90

### Secondary outcomes

1. Clinical:
  a. Weight gain (moderate to good: >5 g/kg/day)
  b. *De novo* development of diarrhoea (>3 loose stools/day)
  c. Time to diarrhoea resolution (if >3 loose stools/day)
  d. Time to bi-pedal oedema resolution (for those with Kwashiorkor)

2. Changes in intestinal biomarkers:
  a. Intestinal cell injury (faecal calprotectin)

Supportive biological and physiological data to support end points will include:

A. Anthropometric:
  a. Weight/height/BMI
B. Microbiota:
  a. Percentage change in relative populations of gut microbiota
C. Metabolomics:
  a. Changes in generation of short chain fatty acids
  b. Changes in host and microbiota metabolic products
D. Lactose intolerance:
  a. Evidence of stool reducing substances at baseline

### Safety reporting

All relevant adverse events will be reported in the case report form (CRF) and serious adverse event (SAE) forms ( Supplementary File 3). The reporting procedure will be captured within a dedicated safety reporting SOP. At each clinical review the child will be assessed for adverse events, both expected and unexpected. Non-serious and expected events will be routinely captured in the CRF. 

### SAEs

SAEs will be reported immediately to the on-site principal investigator (PI) so that co-PIs may be notified of the event. Serious adverse events include:

1. Any untoward medical occurrence or effect that is: (i) fatal, (ii) life threatening, (iii) permanently or temporarily disabling or incapacitating, (iv) causes prolongation of hospital stay ( *if in the clinician’s judgment the adverse event causes the child to stay in hospital longer than would have been necessary if the adverse event had not occurred*.) or (v) any other event that investigator considers serious, having a real, not hypothetical, risk of one of the previous outcomes.

2. Allergic reactions, which will automatically be considered as SAEs in this pilot study.

A dedicated SAE form will be completed including details of the nature of event, date of onset, severity, corrective therapies given, outcome and causality (i.e. unrelated, unlikely, possible, probably, definitely) (see Table 2). This data will be reviewed as part of the study monitoring process.

**Table 2.** Assessment of adverse event.

Parameter	Grade 1 mild	Grade 2 moderate	Grade 3 severe	Grade 4 life-threatening/fatal
Estimating severity grade				
Clinical adverse event NOT identified elsewhere in the grading table	Symptoms causing no or minimal disturbance with current illness	Symptoms causing greater than minimal disturbance with current illness	Symptoms requiring corrective medical treatment	Symptoms requiring medical treatment to prevent permanent impairment, persistent disability, or death
Allergic reaction				
**Acute systemic** **allergic reaction**	Local urticaria or flushing with no medical intervention indicated	Allergic reaction: Fever, chills, flushing, limited pruritic rash, nausea and vomiting without generalised angiooedema or bronchospasm	Grade 2 reaction PLUS Generalized urticaria OR angioedema with medical intervention indicated OR Symptomatic mild bronchospasm	Grade 3 reaction PLUS Acute anaphylaxis OR Life- threatening bronchospasm OR laryngeal edema

### Trial monitoring

This Phase II will be monitored locally, by designated GCP trained and experienced monitors, through assessment of any SAE forms and accumulation of data collected. There is no Data and Safety Monitoring Committee and no planned interim analyses.

### Data management and statistical analysis

Information will be stored securely and will only be made available to those caring for the child and those directly involved in the study. Any reports will not mention the names of the child or parent/guardian. Patient admission data collection forms and observation charts will be kept in a locked cupboard. Designated members of the research team will transcribe relevant data from the source documents to the CRF. Data entry will be done through OpenClinica, an FDA-approved, web-based application, to a relational PostgreSQL database. Security is enforced through authentication of users by use of encrypted passwords. Different access level accounts authorize users on actions they may perform on the database.

Data will be analysed on an intention-to-treat basis. Differences in outcome measures between the three treatment groups will be compared with ANOVA or linear regression as appropriate. Where variables are not normally distributed, they will be transformed by taking logarithms. If this is insufficient, then equivalent non-parametric tests will be used. Metabolomic data will be analysed using multivariate statistical techniques including principal components analysis, projection to latent structures (PLS) and orthogonal PLS.

### Ethical statement

Ethical and regulatory approval has been granted by Imperial College Research Ethics Committee (17IC4146), the sponsor of the study and from Mbale Regional Hospital Institutional Review Committee (019/2018) and UNCST (HS2391). The MIMBLE2 study has been registered on ISRCTN (registry identifier 10309022).

### Safety

There are very few risks attached to this study. Intolerance or allergy to chickpeas are rare. Details of the processing and packaging will be addressed by due diligence of the experienced collaborator (Campden BRI).

Blood samples are required as part of this study; this includes routine blood sampling according national guidelines and an additional 12 ml over the whole duration of the study. Required volumes of blood will be minimized wherever possible and be within the locally agreed maximum. Urine and stool will be collected in a non-invasive manner thus should not cause any distress to the child or their family.

Potential benefit to the child and/or family include:

• Closer observation during admission, which consequently may allow the clinical team to make-important changes to the child’s treatment during in hospital stay.

• All routine non-study medications required by the hospital to treat the child will be made available. Any additional procedure required due to a complication or a potential complication will be covered by the study.

• The parents or guardians for the children will be asked to return for follow up at 28 days after admission. This additional visit will include a clinical review thus an opportunity for medical treatment if required. Education regarding nutrition will also be readdressed at this time. Reimbursement for transport cost for this follow-up visit plus any treatment costs required during the visits will be made.

### Protocol version changes

Version 1. Previous versions approved the use of locally sourced legumes which could not be certified to the same standards as those in the UK.

Version 2. First version included another recruitment site, which became unfeasible.

We do not anticipate any changes to the design of the trial; however, if there were need to alter the trial (e.g., a request for an extension in enrolment, changes or addition of a trial centre and/or investigators), this would be communicated to the ICREC, MRRHREC and UNCST via a formal request for a protocol amendment, as would any other changes in the trial protocol.

## Discussion

Prior to this study we conducted a proof-of-concept study in East Africa in undernourished children MIMbLe Study (Pan African Clinical Trial Registry: PACTR201805003381361) using locally developed cowpea flour added to standard nutritional feeds. We were reassured by the safety and tolerability of this feed. In addition, cowpea-enhanced feeds resulted in a positive effect on increasing bacterial diversity and a favourable decrease in anorectic gut hormones. This led to further funding to enable product development and testing in a randomised control trial. Initially cowpea was targeted as a locally available legume that contains adequate amounts of the resistant starch of interest to promote gut microbial health. The legume-based interventions we propose are used within the local diets but have been milled/processed in such a way to maximise their ability to ferment within the bowel to create a favourable microbiome. The details of the manufacturing process and details of the final product will be available on request (manuscript in preparation).

During the 6-month period of product development we encountered two significant barriers to utilising cowpeas in the current study. First, on testing, a final cowpea-enriched feed product was found to have significantly less resistant starch than anticipated, and second we were not able to identify a commercial supplier whose products met the strict food safety standards required with respect to certification for contaminants including pesticides, toxins, microbes and metals. We therefore opted to use another locally available legume alternative, the chickpea, although this is eaten less frequently in Uganda. On testing we found that the final nutritional paste retained its resistant starch content throughout processing, moreover chickpea (gram) flour was readily available from reputable suppliers. Overall, chickpea production in Uganda in 2016 was estimated to be 5085 tonnes in 2016 by the Food and Agricultural Organization of the United Nations, increasing by approximately 100 tonnes (2.2%) each year since 2010. The safety is assured as intolerance or allergy to chick peas are rare. The G6PD variant in these populations retains >12% of its activity, thus not rendering G6PD-deficient patients to susceptibility to oxidant stress (as seen in Mediterranean variants rendering them susceptible to ‘Favism’) ^23^.

### Potential future impact

An increasing awareness that malnourished populations have higher levels of gut dysfunction with frequent episodes of infection, and a focus on both carbohydrate and protein requirement is inescapable. Enhancing carbohydrate delivery to the colon offers a solution to supporting a mature and diverse microbiome whilst simultaneously promoting gut integrity through SCFA production, together with improving plant-based protein digestibility and bioavailability are both relevant in any attempt to promote greater production and consumption of pulses. Enhancing these qualities in legumes thus offers affordable, scalable and enhanced health management to a very large number of undernourished children. The current phase II trial, if successful, will generate clinical and biological endpoints that will help inform the design and sample size of a future phase III trial with mortality as the primary endpoint.

### Study progress

The modified feed was developed between August 2017 and March 2018. The final product has passed a full microbiological sterility testing following a pre-incubation at 30°C for 14 days, and an incubation on different culture media at 25°C, 37°C and 55°C for a further 7 days. The product is also confirmed to contain <40 µg/kg acrylamide, <400 HAU/g lectin, and <50 mg/kg tin, and was transported to Uganda in June 2018. 

### Recruitment status

The trial started enrolment on 6 ^th^ July 2018 and will recruit over a period of 4–6 months. Primary data analysis and report-writing will take a further 4–6 months.

### Contact information

The contact at Imperial College, for the MRC CIC grant scheme is confidenceinconcept@imperial.ac.uk. Contact for public enquiries should be made to Charles Bernard Okalebo, Mbale Clinical Research Institute, Pallisa Road, PO Box 1966, Mbale, Uganda ( okabecha@gmail.com).

## Data availability

All data underlying the results are available as part of the article and no additional source data are required.

## Supplementary material

**Supplementary File 1. The consent form to be singed/marked by the parent/guardian of each child.**

**Supplementary File 2. Study information given to the parent/guardian of each child.**

**Supplementary File 3. The complete set of case report forms used in the MIMBLE 2.0 study.**
