# Supplementary material for: Modifying Intestinal Integrity and Micro Biome in Severe Malnutrition with Legume-Based Feeds (MIMBLE 2.0): protocol for a phase II refined feed and intervention trial
Source: Wellcome Open Res. 2018 Aug 2;3:95. [Version 1] doi: 10.12688/wellcomeopenres.14706.1 (PMC6171552; doi:10.12688/wellcomeopenres.14706.1)
Supplement: Supplementary file 1 [file wellcomeopenres-3-16015-s0000.tgz › 57ca96b4-9481-4588-bf03-8c5b7dfa7193.docx]

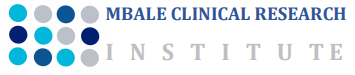
Plot 29-33, Pallisa Road, Mbale (U) | Phone: +256454660416 | Email: info@mcri.ac.ug

Study Consent Form

| Child’s Initials |  |  |  | Study Number |  |  |  |  |  |  |
| --- | --- | --- | --- | --- | --- | --- | --- | --- | --- | --- |

MIMBLE 2.0 (Modifying intestinal integrity and microbiome in severe malnutrition with legume-based feeds): Refined feed and interventions study

Please initial (or mark) box if you agree:

| I confirm that I have read / been read the patient information sheet (version 3.0 dated 16/04/2018) for the MIMBLE 2.0 study and that I understand what will be required if my child participates in the study. The study has been explained to me and my questions have been answered. |  |
| --- | --- |
| I understand that my child’s participation is voluntary and that I am free to withdraw him or her at any time, without giving any reason, without my medical care or legal rights or my child’s medical care or legal rights being affected. |  |
| I understand that sections of any of my child’s medical notes may be looked at by responsible individuals involved in the running of the study or from regulatory authorities where it is relevant to my child’s participation in this research. I give permission for these individuals to have access to my child’s records, but understand that strict confidentiality will be maintained. |  |
| I understand that my child will be invited for a follow-up visit at day 28 and day 90 however will not be discharged with any medication related specifically to the study. After the study, my child’s healthcare will be provided by the national health system. |  |
| I understand that there is no additional financial reimbursement except for treatment/investigation costs during the study and return transport cost from my home for the follow-up visit at day 28 and day 90 |  |
| I agree to allow blood, urine and stool samples to be taken from my child and for my child’s samples to be stored for later testing. I understand that my child and I may not be given the results of tests performed on stored samples. |  |
| I agree to samples being exported overseas for further studies |  |
| I agree for my child to participate in the MIMBLE 2.0 study |  |

| Parent/carer’s signature  (or thumbprint) | Print name | Date (day/month/year) Time |
| --- | --- | --- |
|  |  |  |

| Witness’s signature  (if thumbprint used above) | Print name | Date (day/month/year) Time |
| --- | --- | --- |
|  |  |  |

| Doctor’s signature | Print name | Date (day/month/year) Time |
| --- | --- | --- |
|  |  |  |

IMPORTANT: one signed original to be kept in MIMBLE 2.0 study file by the researcher, one signed copy to be given to the patient, and one signed copy to be kept in the clinic file.
