# Supplementary material for: Modifying Intestinal Integrity and Micro Biome in Severe Malnutrition with Legume-Based Feeds (MIMBLE 2.0): protocol for a phase II refined feed and intervention trial
Source: Wellcome Open Res. 2018 Aug 2;3:95. [Version 1] doi: 10.12688/wellcomeopenres.14706.1 (PMC6171552; doi:10.12688/wellcomeopenres.14706.1)
Supplement: Supplementary file 2 [file wellcomeopenres-3-16015-s0001.tgz › 609eb116-5ae7-4e55-b8de-870a40a8587e.docx]

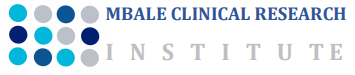
P

Plot 29-33, Pallisa Road, Mbale (U) | Phone: +256454660416 | Email: info@mcri.ac.ug

MIMBLE 2.0 (Modifying intestinal integrity and microbiome in severe malnutrition with legume-based feeds): refined feed and intervention study

Information for Parents and Carers

**A: Introduction**

We are inviting your child to take part in a research study being conducted in this hospital. We will include children between the ages of 6 months to 5 years and aim to involve approximately 160 children over the next 6 months. Before you decide if you want your child to take part, it is important for you to understand why the research is being done and what it will involve. Please take time to read this information sheet carefully or ask someone to read it to you. If there is anything that is unclear or you would like more information please discuss with the nurses or doctors. Joining the study is entirely voluntary. Your decision will not influence the care you receive now or in future.

**B: Study purpose: What is the reason for doing this study?**

Your child has been admitted to the hospital because they have severe acute malnutrition. This is a serious illness and a common problem. We know that in malnutrition the gut (where the food we eat is processed) becomes damaged and does not function properly. This means lots of the food is lost in the stool (poo), sometimes as diarrhoea, and not enough food gets into the body. We all have good bacteria (bugs) living harmlessly in the gut that usually keep us well. However, in malnutrition the types of bacteria living in the gut may change to bad bacteria (bugs) which can lead to serious infection and can cause children to become very ill or die.

The Uganda Ministry of Health, UNICEF and WHO recommend special feeds (F75/F100) for children with malnutrition to increase the amount of energy and nutrients they receive so that they can recover, gain weight and grow. As these feeds are made from milk products they contain a common milk sugar called lactose. Normally this does not cause any problem, however due to damage to the gut, this sugar may not be digested well. If lactose is poorly digested it can make diarrhoea worse, making it more difficult for the child to recover. However, if the gut is damaged and has bad bacteria, and lactose is not digested, then these standard nutritional feeds may not work well.

The aim of this study is to see whether giving children an altered nutritional feed helps repair the gut and encourage the healthy good bacteria, which may reduce illness. In the study, the standard nutritional feed that all children with severe malnutrition receive routinely will be altered by removing the lactose and adding chickpea flour. Chickpea flour could help the good bacteria (bugs) in the gut, and also repair the gut. We want to do this study to find the best nutritional treatment for children with severe malnutrition.

**C: Study Procedures: What will it involve for my child?**

**Study Treatments:**

The doctors will treat your child according to the standard Ugandan Ministry of Health/World Health Organisation (WHO) guidelines for severe acute malnutrition. In the study, all enrolled children will be randomly allocated to receive one of two liquid nutritional feeds at regular times whilst in hospital:

1. Standard WHO (World Health Organisation) feed called F75 or F100.

**OR**

1. A version of F75 or F100 which has lactose removed and chickpea flour added.

You will not know which feed your child is receiving; the decision as to which feed your child gets is decided by a system based on chance, using a computer. When your child is in hospital he or she will only be able to have the feed provided by the hospital.

**Study Procedures:**

1. **What will happen to my child on admission?** As part of usual care when your child is admitted they will be checked by a nurse and doctor, have various measurements (like weight) taken and have routine blood tests. These will help us assess how ill your child is and whether they need additional treatments like a blood transfusion or antibiotics. From this first blood test, we will require some extra blood (1 tablespoon) for the study to store for special tests of your child’s gut health. At this time we will also collect urine and stool (poo).
2. **What measurements will my child have?**  Every day during admission your child will be checked, weighed, have their feed details and amount of urine and stool (poo) produced recorded. We will also do some extra quick measurements to assess growth.
3. **What extra tests will my child have?** On day 7, day 28 and day 90 we will collect blood, urine and stool (poo) samples to look at how healthy the gut is and the bacteria (bugs). Blood samples will be taken at the same time as routine blood tests.
4. **What will happen to the samples that are collected?** Most of the samples will be frozen and stored as the majority of the tests require specialised techniques not available in Uganda. The samples will be sent to a laboratory abroad to do this testing. However, this will happen after you finish the study, so you will not be given the results of these tests.
5. **How long will the study continue?** The study will continue for 90 days. The time that your child remains in hospital is decided by national guidelines so you will go home at the same time whether your child is in the study or not. However, we will ask your child to return for the day (2 hours) 28 days and 90 days after they are first admitted to have a check-up and some tests.

**D: Risks of study participation**

There are very few risks to your child being in this study. The chickpea is milled (made into flour) in the UK, and added to the standard ingredients used to make the nutritional milks advised by the WHO. The only other change is that the ingredients used will be lactose free. The total amount of blood taken during the study will not harm the health of your child. All children will also have urine and stool (poo) collected; this should not cause any distress to your child. If for any reason the doctor thinks that it is not in your child’s best interests to be in the study then they will not be enrolled in the study but will be given their usual treatment.

**E: Benefits of study participation**

Your child will get no direct benefits from this study. However, your child will get close observation which will enable us to make important changes to their treatment if needed. You will be asked to bring your child back for a follow up visit during which we will treat any illnesses we find or arrange referral to an appropriate clinic or hospital. Routine medicines and medical tests we perform during the study will be paid for by the study. Additionally, by taking part your child may help us improve the care of children who have malnutrition in the future.

**G: Compensation**

It will not cost you any money to take part in this study. All your travel expenses for attending the day 28 and day 90 follow-up visits will be paid based on the cost of public transport to and from your home. This will be according to national recommendations: UGX 10,000 to 20,000 depending on location.

**H: Confidentiality. Who will have access to information about me/my child in this research?**

All research records are stored securely in locked cabinets and password protected computers. Only a few people who are working closely on the study will be able to view information from your child. No reports of the study will include any information that will make it possible to identify your child.

**I: Study related injury**

This research is supported by Imperial College London who holds insurance policies which apply to this study. In case of any study related injury, the study will provide care until complete cure or stabilization of the research participant and the best care within the country will be given to your child. If you would like more specific information related to this please discuss it with our staff.

**J: Study regulation: Who has allowed this research to take place?**

This research study has been approved by both local Soroti and Mbale Regional Referral Hospitals and Imperial College London ethics committees.

**K: Further Questions: What if I have any questions?**

You may ask any of our staff questions at any time or contact those who are responsible for the care of your child and this research:

**Dr Peter Olupot Olupot; MBCHB, MPH, PHD;** Mbale Regional Referral Hospital, P.O Box 921, Mbale. [polupotolupot@yahoo.com](mailto:ctegu@yahoo.com); Mobile : + 256 (0)772457217/ Tel: (0)392910171/(0)352280584/(0)45 4433193 Fax: +256 45 4435894

If you want to ask someone independent about this research please contact:

*The Chairman, Mbale Regional Referral Hospital Research & Ethics Committee (MRRH-REC), Dr. John Stephen Obbo Olwenyi, P.O. Box 921, Mbale, Uganda. Telephone 0772 437 407.*

Before you sign this form, please ask any questions on any aspect of this study that is unclear to you. You may take as much time as necessary to think it over.
