# Supplementary material for: Modifying Intestinal Integrity and Micro Biome in Severe Malnutrition with Legume-Based Feeds (MIMBLE 2.0): protocol for a phase II refined feed and intervention trial
Source: Wellcome Open Res. 2018 Aug 2;3:95. [Version 1] doi: 10.12688/wellcomeopenres.14706.1 (PMC6171552; doi:10.12688/wellcomeopenres.14706.1)
Supplement: Supplementary file 3 [file wellcomeopenres-3-16015-s0002.tgz › a3789167-4210-4247-8daa-8a06b866a8c2.pdf]

Study ID Number: *(add post randomisation)*		Patient Initials:				Hospital Number:
MIM2_ [ ] [ ] [ ]						[ ][ ][ ][ ][ ][ ][ ][ ][ ]

SCREENING, ELIGIBILITY , CONSENT AND RANDOMISATION	
Child's name	
Date of birth	D D M M Y Y Y Y
Age (years/months)	Y Y M M
Gender	[ ] Male [ ] Female
Admission date	D D M M Y Y Y Y
Screening date *(if different from admission)*	D D M M Y Y Y Y

SCREENING AND ELIGIBILITY INFORMATION *Please complete the MIMBLE 2.0 screening log for all patients*			
MUAC (cm)	[ ][ ].[ ]cm	MUAC <11.5cm	[ ] Yes [ ] No
Oedema Score	0 + ++ +++	Oedema Location (if present)	[ ] Pre-tibial<br>[ ] Arms and legs<br>[ ] Generalised

INCLUSION CRITERIA (ALL MUST BE YES)	
Age between ≥6 months ≤ 5 years (60 months)	[ ] Yes [ ] No
Does the child have marasmus (MUAC <11.5cm) or kwashiorkor (bilateral pitting oedema)?	[ ] Yes [ ] No
Is the child/parent willing to consent to participate in the study?	[ ] Yes [ ] No
**EXCLUSION CRITERIA (ALL MUST BE NO)**	
High risk of death due to comorbidity	[ ] Yes [ ] No
Terminal illness	[ ] Yes [ ] No

CONSENT	
Is the child appropriate for inclusion based on screening/eligibility?	[ ] Yes [ ] No *if yes proceed to consent*
Was written consent obtained before randomisation?	[ ] Yes [ ] No
Date and Time of consent	D D M M Y Y Y Y Hr Hr min min
If consent is delayed, has verbal assent been obtained?	[ ] Yes [ ] No

RANDOMISATION			
Time (24 hour) of randomisation	Hr Hr min min	Treatment strategy	[ ] WHO feeds (control)<br>[ ] Trial feeds (intervention)
Study ID Number	MIM2_[ ][ ][ ]		

FORM COMPLETED BY		
Name (Printed)	Signature	Date
		D D M M Y Y Y Y

Study ID Number:		Patient Initials:				Hospital Number:
MIM2_ [ ] [ ] [ ]						[ ][ ][ ][ ][ ][ ][ ][ ]

BASELINE DETAILS

Admission Clinical Assessment

Clinical history of current illness		
History of fever	☐ Yes	☐ No
History of fever for more than 14 days	☐ Yes	☐ No
History of cough	☐ Yes	☐ No
Increased work of breathing	☐ Yes	☐ No
Diarrhoea *(>3 watery stools in 24 hours)*	☐ Yes	☐ No
Passing worms in stools	☐ Yes	☐ No
Vomiting	☐ Yes	☐ No
Fits in this illness	☐ Yes	☐ No

Recent and current medical treatment			
Admitted for over 24 hours in another facility		☐ Yes	☐ No
Is participant currently taking antibiotics?		☐ Yes	☐ No
Duration of treatment to date (days)		[ ][ ]	
If yes, please tick box any of antibiotic belows (can be more than one):			
☐ Amoxicillin	☐ Co-trimoxazole	☐ Metronidazole	☐ Does not know name
☐ Cephalixin or similar ☐ Other- please specify:			
Has the child taken oral antibiotics in last one month?		☐ Yes	☐ No
Is participant on anti-malarial medication currently?		☐ Yes	☐ No
Is participant on anti-retroviral medication currently?		☐ Yes	☐ No
Is participant currently receiving traditional medicines?		☐ Yes	☐ No
If yes, please specify:			

Chronic conditions present at enrolment		
TB (on treatment)	☐ Yes	☐ No
Chronic cough (> 1month)	☐ Yes	☐ No
Has the participant had diarrhoea in the last 6 months?	☐ Yes	☐ No
If yes to diarrhoea in the last 6 months, which of the below options would best describe the diarrhoea?		
☐ Short episode (<1 week)	☐ Recurrent	☐ Persistent
Sickle cell anaemia	☐ Yes	☐ No
Dark urine syndrome	☐ Yes	☐ No

Completed by: Study Doctor (Name)	Signature	Date
		DD/MM/YYYY

Study ID Number:		Patient Initials:				Hospital Number:
MIM2_ [ ] [ ] [ ]						[ ][ ][ ][ ][ ][ ][ ][ ][ ]

Congenital or acquired heart disease	☐ Yes	☐ No
Cerebral palsy or severe developmental delay	☐ Yes	☐ No
Epilepsy	☐ Yes	☐ No
Other (specify):		

**Feeding History**			
Age food was introduced	| _ | | _ | months		
Is the participant still breastfeeding at all?	☐ Yes	☐ No	
Was the participant born prematurely?	☐ Yes	☐ No	☐ Don't know

**Malnutrition History**			
Has the participant EVER been admitted to hospital for malnutrition before?	☐ Yes	☐ No	☐ Don't know
In this illness, was the participant admitted to another hospital?	☐ Yes	☐ No	☐ Don't know
Is participant currently on RUTF?	☐ Yes	☐ No	☐ Don't know
Ask the parent/guardian to rate the child's appetite (0 = Poor, 10 = Very Good):	☹ 0 1 2 3 4 5 6 7 8 9 10 ☺		

**Developmental milestones**		
Was the participant able to sit unsupported before this illness?	☐ Yes	☐ No
Was the participant able to walk unsupported before this illness?	☐ Yes	☐ No

Completed by: Study Doctor (Name)	Signature	Date
		DD/MM/YYYY

Study ID Number:		Patient Initials:				Hospital Number:
MIM2_ [ ] [ ] [ ]						[ ][ ][ ][ ][ ][ ][ ][ ]

Admission Clinical Examination

Chest				
In-drawing			☐ Yes	☐ No
Deep breathing			☐ Yes	☐ No
Crackles	☐ None	☐ Unilateral	☐ Bilateral	
**Abdomen**				
Severe palmar pallor			☐ Yes	☐ No
Jaundice			☐ Yes	☐ No
Liver size			[ ][ ] cm	
Ascites			☐ Yes	☐ No
Splenomegaly			☐ Yes	☐ No
**Dermatosis**				
Flaky paint skin			☐ Yes	☐ No
Desquamation (peeling)			☐ Yes	☐ No
**Mouth**				
Candida			☐ Yes	☐ No
Angular kelosis			☐ Yes	☐ No
Mouth sores			☐ Yes	☐ No
**Eyes**				
Conjunctivitis			☐ Yes	☐ No
Corneal changes			☐ Yes	☐ No
**Ears**				
Otitis externa			☐ Yes	☐ No
Otitis media			☐ Yes	☐ No
**Lymphadenopathy**				
Generalised lymphadenopathy			☐ Yes	☐ No

Completed by: Study Doctor (Name)	Signature	Date
		DD/MM/YYYY

Study ID Number:		Patient Initials:				Hospital Number:
MIM2_ [ ] [ ] [ ]						[ ][ ][ ][ ][ ][ ][ ][ ][ ]

Working Diagnosis	
To be completed within 48 hours; please <u>pick all relevant diagnoses</u>.	
Malaria (*confirmed by slide or RDT positive)*	☐ Yes
Profound anaemia (Hb < 4 g/dL)	☐ Yes
Gastroenteritis (> 3 watery stools in 24 hours)	☐ Yes
Helminth infection	☐ Yes
LRTI- all types	☐ Yes
Tuberculosis	☐ Yes
Congenital Heart Condition	☐ Yes
HIV/AIDS	☐ Yes
Sepsis/Septicaemia	☐ Yes
Meningitis	☐ Yes
Encephalopathy	☐ Yes
Cerebral palsy	☐ Yes
Osteomyelitis/Pyogenic Arthritis	☐ Yes
Skin infection	☐ Yes
Urinary tract infection	☐ Yes
Pyrexia of unknown infection	☐ Yes
Sickle cell anaemia	☐ Yes
Dark urine syndrome	☐ Yes
Other 1 (please provide details):	
Other 2 (please provide details):	
Other 3 (please provide details):	
Other 4 (please provide details):	

Completed by: Study Doctor (Name)	Signature	Date
		DD/MM/YYYY

Study ID Number:		Patient Initials:				Hospital Number:
MIM2_ [ ] [ ] [ ]						[ ][ ][ ][ ][ ][ ][ ][ ][ ]

Admission Clinical testing

Point of Care Ward Test Results				
**Test**	**Result**			**Not done**
Urinalysis	***Please complete CRF 6C***			☐
Glucose	[ ][ ].[ ][ ] mmol/L			☐
Lactate	[ ][ ].[ ][ ] mmol/L			☐
HIV	☐ Positive	☐ Negative	☐ Invalid	☐
Malaria Rapid DT	☐ Positive	☐ Negative	☐ Invalid	☐

ADMISSION SAMPLES				
Sample Type:	Volume:	Collected?	Real time test/ storage:	Form to complete
EDTA (purple top)	1 x 0.5ml	[ ] Yes / [ ] No	Malaria and FBC	CRF 5<br>Source Doc 5
Blood culture (BACTEC)	2ml	[ ] Yes / [ ] No	Microbiology	CRF 6A<br>Source Doc 5
Lithium Heparin (green top)	1 x 4ml	[ ] Yes / [ ] No	Biochemistry	CRF 6B<br>Source Doc 5
Urine	1 pot (5mls)	[ ] Yes / [ ] No	Urinalysis	CRF 6C
Faeces	1 pot (10g)	[ ] Yes / [ ] No	Microscopy	CRF 6A

Completed by: Study Nurse (Name)	Signature	Date
		DD/MM/YYYY

Study ID Number:		Patient Initials:				Hospital Number:
MIM2_ [ ] [ ] [ ]						[ ][ ][ ][ ][ ][ ][ ][ ][ ]

VISIT DETAILS

Study follow up visit	[ ] Day 1	[ ] Day 7	[ ] Day 28	[ ] Day 90
Date of follow up visit	DD/MM/YYYY	Did the child attend the visit?	[ ] Yes [ ] No	
Was the child an inpatient at the visit?	[ ] Yes<br>[ ] No	Are contact details still correct?	[ ] Yes [ ] No<br>*if no please complete new Source Doc 5*	

CLINICAL DETAILS

A: Physical Examination at follow-up

Weight (kg)	[ ][ ].[ ]kg	Height/length (cm)	[ ][ ][ ].[ ]cm
MUAC (cm)	[ ][ ].[ ]cm	MUAC <11.5cm	[ ] Yes [ ] No
Oedema Score	[ ] None<br>[ ] Pretibial<br>[ ] Hands/legs<br>[ ] Generalised		

Ask the parent/guardian to rate the child's appetite (0 = Poor, 10 = Very Good):

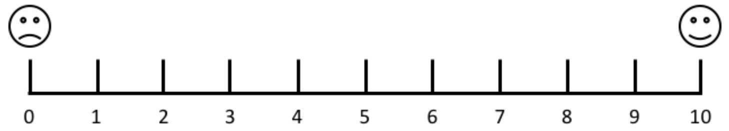

B: Symptoms at follow-up

i.	Fever	[ ] Yes[ ] No	viii.	Abdominal aching/pain	[ ] Yes[ ] No
ii.	Weight loss	[ ] Yes[ ] No	ix.	Poor appetite	[ ] Yes[ ] No
iii.	Severe pallor	[ ] Yes[ ] No	x.	Sore mouth/throat/ulcers/thrush	[ ] Yes[ ] No
iv.	Cough	[ ] Yes[ ] No	xi.	Diarrhoea	[ ] Yes[ ] No
v.	If yes, for more than 14 days	[ ] Yes[ ] No<br>[ ] NA	xii.	Other (specify): ______________	[ ] Yes[ ] No
vi.	Difficulty breathing	[ ] Yes[ ] No	xiii.	Other (specify): ______________	[ ] Yes[ ] No
vii.	Lethargy	[ ] Yes[ ] No	xiv.	Other (specify): ______________	[ ] Yes[ ] No

D: Medication (list any medications prescribed or taken since last MIMBLE 2.0 visit)

Medication	Reason Prescribed	Dose	Units (e.g. mg)	Frequency	Date of prescription	No of days prescribed
					D D M M Y Y Y Y	
					D D M M Y Y Y Y	
					D D M M Y Y Y Y	
					D D M M Y Y Y Y	
					D D M M Y Y Y Y	
					D D M M Y Y Y Y	

Study ID Number:	Patient Initials:	Hospital Number:
MIM2_ [ ] [ ] [ ]		[ ][ ][ ][ ][ ][ ][ ][ ][ ]

CLINICAL EVENT

Has the child had any of the following since the last MIMBLE 2.0 review?

1. Adverse event — [ ] Yes [ ] No (*if yes, add details below using codes at end of this form and complete adverse event/serious adverse event CRF 9)*

Description	Onset date	End date	Severity	Causality	Outcome	SAE	Initials
	DD/MM/YYYY	DD/MM/YYYY	[ ]	[ ]	[ ]	[ ] Yes<br>[ ] No	
	DD/MM/YYYY	DD/MM/YYYY	[ ]	[ ]	[ ]	[ ] Yes<br>[ ] No	
	DD/MM/YYYY	DD/MM/YYYY	[ ]	[ ]	[ ]	[ ] Yes<br>[ ] No	
	DD/MM/YYYY	DD/MM/YYYY	[ ]	[ ]	[ ]	[ ] Yes<br>[ ] No	

2. Received antibiotics since the last visit? — [ ] Yes [ ] No *if yes add details to section D above*

3. Diarrhoea

Has the child had diarrhoea since the last review?	[ ] Yes [ ] No
If yes, when did it start?	[DD]/[MM]/[YYYY]
Did the diarrhoea resolve?	[ ] Yes [ ] No
If yes, when did it resolve?	[DD]/[MM]/[YYYY]

FOLLOW UP SAMPLES

Sample type:	Volume:	Collected?	Real time test/storage	Form to complete
EDTA (purple top)	1 x 0.5ml	[ ] Yes / [ ] No	Malaria and FBC	CRF 5<br>Source Doc 5
Blood culture (BACTEC)	2ml	[ ] Yes / [ ] No	Microbiology	CRF 6A<br>Source Doc 5
Lithium Heparin (green top)	1 x 4ml	[ ] Yes / [ ] No	Biochemistry	CRF 6B<br>Source Doc 5
	1 x 4ml	[ ] Yes / [ ] No	Storage	Source Doc 4<br>Source Doc 5
Urine	1 pot (5mls)	[ ] Yes / [ ] No	Urinalysis	CRF 6C
	1 pot (5mls)	[ ] Yes / [ ] No	Storage	Source Doc 4<br>Source Doc 5
Faeces	1 pot (10g)	[ ] Yes / [ ] No	Microscopy/Storage	CRF 6A<br>Source Doc 4<br>Source Doc 5

Completed by: Name	Signature	Date

Adverse event severity code	Causality	Outcome
1. Mild<br>2. Moderate<br>3. Severe<br>4. Life threatening	1. Definitely<br>2. Probably<br>3. Possibly<br>4. Unlikely<br>5. Unrelated<br>6. Not assessable	1. Resolved<br>2. Resolved with sequelae<br>3. Unresolved at discharge<br>4. Fatal<br>5. Unknown

24-HOUR DIETARY RECALL CRF

Study ID Number:		Patient Initials:				Hospital Number:
MIM2_ [ ] [ ] [ ]						[ ][ ][ ][ ][ ][ ][ ][ ]

	**Time** (24h clock)	**Name of food or drink**	**Preparation**	**Portion** *(if estimated by weight from item)*	**Volume (ml)** *(if estimated using weight from volume)*	**Weight (g)***
**Item 1**						
**Item 2**						
**Item 3**						
**Item 4**						
**Item 5**						
**Item 6**						
**Item 7**						
**Item 8**						
**Item 9**						
**Item 10**						
**Item 11**						
**Item 12**						
**Item 13**						
**Item 14**						
**Item 15**						
**Item 16**						
**Item 17**						
**Item 18**						
**Item 19**						
**Item 20**						
**Item 21**						
**Item 22**						
**Item 23**						
**Item 24**						
**Item 25**						
**Item 26**						

*If food portion size is not known/remembered by the patient enter ‘8888’ in ‘Weight (g)’ column
*If food portion size was no requested by the assessor enter ‘9999’ in ‘Weight (g)’ column

Completed by: Name	Signature	Date
		DD/MM/YYYY

24-HOUR DIETARY RECALL CRF

Study ID Number:		Patient Initials:				Hospital Number:
MIM2_ [ ] [ ] [ ]						[ ][ ][ ][ ][ ][ ][ ][ ]
Study Visit	[ ] Admission		[ ] Day 28			[ ] Day 90

	**Time** (24h clock)	**Name of food or drink**	**Preparation**	**Portion** *(if estimated by weight from item)*	**Volume (ml)** *(if estimated using weight from volume)*	**Weight (g)***
**Item 1**						
**Item 2**						
**Item 3**						
**Item 4**						
**Item 5**						
**Item 6**						
**Item 7**						
**Item 8**						
**Item 9**						
**Item 10**						
**Item 11**						
**Item 12**						
**Item 13**						
**Item 14**						
**Item 15**						
**Item 16**						
**Item 17**						
**Item 18**						
**Item 19**						
**Item 20**						
**Item 21**						
**Item 22**						
**Item 23**						
**Item 24**						
**Item 25**						

*If food portion size is not known/remembered by the patient enter ‘8888’ in ‘Weight (g)’ column
*If food portion size was no requested by the assessor enter ‘9999’ in ‘Weight (g)’ column

Completed by: Name	Signature	Date
		DD/MM/YYYY

Study ID Number:		Patient Initials:				Hospital Number:
MIM2_ [ ] [ ] [ ]						[ ][ ][ ][ ][ ][ ][ ][ ]

LABORATORY TEST RESULTS

Sample Details (to be completed in clinic by the person requesting the test)						
Sample Date	DD/MM/YYYY		Sample Time (24hr clock)	HH:MM		
Study time point	[ ] Admission	[ ] Day 1	[ ] Day 7	[ ] Day 28	[ ] Day 90	[ ] Other Specify:______
Requested by:	Name: ___________________________		Signature: ______________________		Date: DD/MM/YYYY	

Haematology (to be completed in the laboratory and sent to clinic)			
Test	Result	Unit (circle units used)	Not Done
WBC	[ ][ ][ ][ ].[ ][ ]	10^3/uL or 10^9/L	[ ]
Lymphocytes	[ ][ ][ ][ ].[ ][ ]	10^3/uL or %	[ ]
Neutrophils	[ ][ ][ ][ ].[ ][ ]	10^3/uL or %	[ ]
RBC	[ ][ ][ ][ ].[ ][ ]	10^6/uL or 10^{12}/L	[ ]
Hb	[ ][ ][ ][ ].[ ][ ]	g/dL	[ ]
Haematocrit	[ ][ ][ ][ ].[ ][ ]	%	[ ]
MCV	[ ][ ][ ][ ].[ ][ ]	fL	[ ]
Platelets	[ ][ ][ ][ ].[ ][ ]	10^3/uL or 10^9/L	[ ]
Granulocytes	[ ][ ][ ][ ].[ ][ ]	10^9/uL or %	[ ]

Malaria Investigations (note malaria investigations on admission only unless clinically indicated)				
Test	Positive	Negative	Invalid	Not Done
Malaria blood film	[ ]	[ ]	[ ]	[ ]
If positive tick all that apply:	[ ] P. falciparum	[ ] P. malariae	[ ] P. ovale	[ ] P. vivax
If positive, parasite load:	[ ][ ][ ][ ][ ][ ]	[ ] Per 200 WBC<br>[ ] Per 500 RBC		

Completed by:	Initials: ____________________	Date:	DD/MM/YYYY

Study ID Number:		Patient Initials:				Hospital Number:
MIM2_ [ ] [ ] [ ]						[ ][ ][ ][ ][ ][ ][ ][ ]

STOOL PARASITOLOGY TEST RESULTS

Stool Microscopy (to be completed in the laboratory and sent to the ward)		
Stool Appearance	[ ] Normal	[ ] Abnormal
	If abnormal, please describe how: ______________________________	
Blood present	[ ] Yes	[ ] No
Parasites	[ ] no parasites	[ ] parasites, ova or cysts seen
Specify organisms identified:		

Completed by:	Initials: ____________________	Date:	DD/MM/YYYY

Study ID Number:		Patient Initials:				Hospital Number:
MIM2_ [ ] [ ] [ ]						[ ][ ][ ][ ][ ][ ][ ][ ]

Study ID Number:		Patient Initials:				Hospital Number:
MIM2_ [ ] [ ] [ ]						[ ][ ][ ][ ][ ][ ][ ][ ][ ]

BATCH BIOCHEMISTRY TEST RESULTS

Sample Details						
Collection Date	DD/MM/YYYY		Collection Time (24hr clock)	HH:MM		
Study time point	[ ] Admission	[ ] Day 1	[ ] Day 7	[ ] Day 28	[ ] Day 90	[ ] Other Specify:_______
Biochemistry (for batch testing): Form to be completed in the laboratory and sent to clinic, or in the clinic using results sent by the laboratory						

Test	Result	Unit	Not Done
Sodium	[ ][ ][ ][ ].[ ][ ]	mmol/L	[ ]
Potassium	[ ][ ][ ][ ].[ ][ ]	mmol/L	[ ]
Urea	[ ][ ][ ][ ].[ ][ ]	mg/dL or mmol/L	[ ]
Creatinine	[ ][ ][ ][ ].[ ][ ]	umol/L or mg/dL	[ ]
Albumin	[ ][ ][ ][ ].[ ][ ]	g/dL or g/L	[ ]
AST	[ ][ ][ ][ ].[ ][ ]	U/L	[ ]
ALT	[ ][ ][ ][ ].[ ][ ]	U/L	[ ]
Bilirubin (Total)	[ ][ ][ ][ ].[ ][ ]	umol/L or mg/dL	[ ]

Completed by:	Initials: ____________________	Date:	DD/MM/YYYY

Study ID Number:		Patient Initials:				Hospital Number:
MIM2_ [ ] [ ] [ ]						[ ][ ][ ][ ][ ][ ][ ][ ]

URINE DIPSTICK TEST RESULTS (Completed on Ward)

Urine dipstick results	[ ] Too dark to read	Hammersmith colour chart number (1-10)	[ ][ ]

Test	Result (Please Circle)					
Glucose (mg/dL)	Neg.		100	250	500	1000	≥2000
Bilirubin	Neg.				Small +	Moderate ++	Large +++
Ketone (mg/dL)	Neg.		Trace 5	Small 15	Moderate 40	Large 80	Large 160
Specific Gravity	1.000	1.005	1.010	1.015	1.020	1.025	1.030
Blood	Non-haemolyzed			Haemolyzed			
	Neg.	Trace	Moderate	Trace	Small +	Moderate ++	Large +++
pH	5.0	6.0	6.5	7.0	7.5	8.0	8.5
Protein	Neg.	Trace		30 +	100 ++	300 +++	≥2000 ++++
Urobilinogen (mg/dL)	Normal 0.2	Normal 1			2	4	8
Nitrite	Neg.					Positive Any form of uniform pink	
Leukocytes	Neg.			Trace	Small +	Moderate ++	Large +++

Completed by:	Initials: ____________________	Date:	DD/MM/YYYY

Study ID Number:		Patient Initials:				Hospital Number:
MIM2_ [ ] [ ] [ ]						[ ][ ][ ][ ][ ][ ][ ][ ]

BLOOD CULTURE RESULTS

Sample Details (to be completed in the laboratory and sent to the ward)						
Collection Date	DD/MM/YYYY	Collection Time (24hr clock)	HH:MM			
Study time point	[ ] Admission	[ ] Day 1	[ ] Day 7	[ ] Day 28	[ ] Day 90	[ ] Other Specify:______
Blood culture (for batch testing): Form to be completed in the laboratory and sent to clinic, or in the clinic using results sent by the laboratory						
Pathogen isolated	[ ] No	[ ] Yes (specify below)				
Hours to positivity	[ ][ ][ ] hours	[ ] N/A				
Isolated pathogen:		[ ] N/A				

Completed by:	Initials:___________________________	Date:	DD/MM/YYYY

Study ID Number:		Patient Initials:		Hospital Number:
MIM2_ [ ] [ ] [ ]				[ ][ ][ ][ ][ ][ ][ ][ ]

A: Discharge details	
How was the child discharged?	[ ] Absconded Date: DD/MM/YYYY<br>[ ] Died Date: DD/MM/YYYY<br>[ ] Discharged home Date: DD/MM/YYYY<br>[ ] Self-discharged home Date: DD/MM/YYYY
How long was the child's admission?	[ ][ ]days

B: Physical Examination at follow-up			
Weight (kg)	[ ][ ].[ ]kg	Height (cm)	[ ][ ][ ].[ ]cm
MUAC (cm)	[ ][ ].[ ]cm	MUAC <11.5cm	[ ] Yes [ ] No
Oedema Score	[ ] None<br>[ ] Pretibial<br>[ ] Hands/legs<br>[ ] Generalised	Did oedema resolve?	[ ] Yes [ ] No<br>If yes, how many days did it take to resolve? [ ][ ]

Diarrhoea on discharge (>3 watery stools in 24hrs)	Does the child have diarrhoea now? [ ] Yes [ ] No<br>If yes, when did it start? [DD]/[MM]/[YYYY]
Did child receive any of the following during admission?	ReSoMal [ ] Yes / [ ] No If yes on how many days: [ ][ ]<br>Transfusion [ ] Yes / [ ] No If yes how many units were given: [ ][ ]<br>IV fluid [ ] Yes / [ ] No If yes on how many days: [ ][ ]

C: Medications on discharge (include any nutrition supplements prescribed)

Medication	Reason Prescribed	Dose	Units (e.g. mg)	Frequency	Date of prescription	No of days prescribed
					D D M M Y Y Y Y	
					D D M M Y Y Y Y	
					D D M M Y Y Y Y	
					D D M M Y Y Y Y	
					D D M M Y Y Y Y	
					D D M M Y Y Y Y	
					D D M M Y Y Y Y	

D: Discharge Checklist: 1. Ensure contact details on Source Document 5 are still correct
2. Ensure that the next study visit appointment has been confirmed with the guardian

Completed by:	Name:_____________________	Date:	DD/MM/YYYY

Study ID Number:		Patient Initials:				Hospital Number:
MIM2_ [ ] [ ] [ ]						[ ][ ][ ][ ][ ][ ][ ][ ][ ]

A: Primary/Secondary outcome measurements			
What study time point did the patient finish the study?	[ ] Day 7 [ ] Day 28 [ ] Day 90<br>[ ] Other, specify ________		
Did child have diarrhoea on admission?	[ ] Yes [ ] No	If yes, has admission diarrhoea resolved by exit from study?	[ ] Yes [ ] No<br>If yes, number of days to improvement [ ][ ]
Did child develop new diarrhoea during hospital admission?	[ ] Yes [ ] No	If yes, has new diarrhoea resolved by exit from study?	[ ] Yes [ ] No<br>If yes, number of days to improvement [ ][ ]
Ask the parent/guardian to rate the child's appetite (0 = Poor, 10 = Very Good):	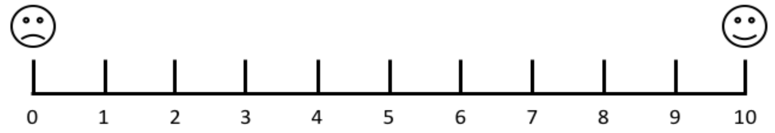		
Weight on exit from study	[ ][ ][ ].[ ]kg		
MUAC (cm) on exit from study	[ ][ ].[ ]cm	Did MUAC recover? (>12.5cm)	[ ] Yes [ ] No
Oedema Score on exit from study	[ ] None<br>[ ] Pretibial<br>[ ] Hands/legs<br>[ ] Generalised	Did oedema resolve?	[ ] Yes [ ] No<br>If yes, number of days to resolution [ ][ ]

Completed by:	Name:_________________________	Date:	DD/MM/YYYY

Study ID Number:		Patient Initials:				Hospital Number:
MIM2_ [ ] [ ] [ ]						[ ][ ][ ][ ][ ][ ][ ][ ]

A: Serious Adverse Events (refer to MOP v1.0 for examples)							
Event Description	Code	Onset Date	Ongoing	End Date	Severity	Outcome	Causality
		DD/MM/YYYY	[ ]	DD/MM/YYYY			
		DD/MM/YYYY	[ ]	DD/MM/YYYY			
		DD/MM/YYYY	[ ]	DD/MM/YYYY			
		DD/MM/YYYY	[ ]	DD/MM/YYYY			
Intervention arm	[ ] Strategy A (WHO Standard of Care) / [ ] Strategy B: Legume-enriched feed						
Narrative of event(s)							

Event Code:			
1 Anaphylaxis	4 Convulsion		7 Death
2 Allergic reaction (any grade)	5 Sepsis		8 Readmission
3 Profuse diarrhoea	6 Shock		9 Other

If allergic reaction please complete:

Grade	Severity	Description	Yes	No
1	Mild	Local urticaria with no medical intervention indicated	[ ]	[ ]
2	Moderate	Allergic reaction: fever, chills, flushing, limited pruritic rash, nausea and vomiting without generalised angioedema or bronchospasm	[ ]	[ ]
3	Severe	Grade 2 reaction PLUS generalised urticaria OR angioedema with medical intervention indicated OR symptomatic mild bronchospasm	[ ]	[ ]
4	Life-threatening	Grade 3 reaction PLUS acute anaphylaxis OR life-threatening bronchospasm OR laryngeal oedema	[ ]	[ ]

Important Information	Adverse event severity code	Causality	Outcome
	1. Mild<br>2. Moderate<br>3. Severe<br>4. Life threatening	1. Definitely<br>2. Probably<br>3. Possibly<br>4. Unlikely<br>5. Unrelated<br>6. Not assessable	1. Resolved<br>2. Resolved with sequelae<br>3. Unresolved at discharge<br>4. Fatal<br>5. Unknown

Completed by:	Name:___________________________	Date:	DD/MM/YYYY

MIMBLE 2.0 | Case Report Form 10: Feed provision | 04/06/2018 | [ ] Control

Study ID Number:		Patient Initials:				Hospital Number:
MIM2_[ ][ ][ ]						[ ][ ][ ][ ][ ][ ][ ][ ]

[ ] Legume-enriched

Day (if inpatient, tick & complete)	Adm [ ]	Day 1 [ ]	Day 2 [ ]	Day 3 [ ]	Day 4 [ ]	Day 5 [ ]	Day 6 [ ]	Day 7 [ ]
Date	DD/MM/YYYY	DD/MM/YYYY	DD/MM/YYYY	DD/MM/YYYY	DD/MM/YYYY	DD/MM/YYYY	DD/MM/YYYY	DD/MM/YYYY
Treatment Phase	☐Stabilise ☐Rehab	☐Stabilise ☐Rehab	☐Stabilise ☐Rehab	☐Stabilise ☐Rehab	☐Stabilise ☐Rehab	☐Stabilise ☐Rehab	☐Stabilise ☐Rehab	☐Stabilise ☐Rehab
Child's weight (kg)	[kg][kg].[kg]	[kg][kg].[kg]	[kg][kg].[kg]	[kg][kg].[kg]	[kg][kg].[kg]	[kg][kg].[kg]	[kg][kg].[kg]	[kg][kg].[kg]
Target ml or g per feed								
If legume-enriched feed, target additional water per feed (ml)								

		Feed	Water	Feed	Water	Feed	Water	Feed	Water	Feed	Water	Feed	Water	Feed	Water	Feed	Water
Time of feed/extra water and amount taken (mls/g)	1: HH:MM																
	2: HH:MM																
	3: HH:MM																
	4: HH:MM																
	5: HH:MM																
	6: HH:MM																
Total ml or g taken																	

	Adm	Day 1	Day 2	Day 3	Day 4	Day 5	Day 6	Day 7
Mark (x) if child vomited after feed	1 2 3 4 5 6	1 2 3 4 5 6	1 2 3 4 5 6	1 2 3 4 5 6	1 2 3 4 5 6	1 2 3 4 5 6	1 2 3 4 5 6	1 2 3 4 5 6
If legume-enriched feed, record the batch number	1 2 3 4 5 6	1 2 3 4 5 6	1 2 3 4 5 6	1 2 3 4 5 6	1 2 3 4 5 6	1 2 3 4 5 6	1 2 3 4 5 6	1 2 3 4 5 6
Any feed missed? Use codes in footnote to record reason	☐ Yes / ☐ No ____________	☐ Yes / ☐ No ____________	☐ Yes / ☐ No ____________	☐ Yes / ☐ No ____________	☐ Yes / ☐ No ____________	☐ Yes / ☐ No ____________	☐ Yes / ☐ No ____________	☐ Yes / ☐ No ____________
Receiving feed via NG?	☐ Yes / ☐ No	☐ Yes / ☐ No	☐ Yes / ☐ No	☐ Yes / ☐ No	☐ Yes / ☐ No	☐ Yes / ☐ No	☐ Yes / ☐ No	☐ Yes / ☐ No
Completed by:								
Initials:								

Footnote: 1 – Unconscious; 2 – Not present on ward at time of feed; 3 – Refused/Dislikes; 4 – Nausea or Vomiting; 5 – Other Reason (specify):________________

Case Report Form 10: Feed provision

04/06/2018

[ ] Control

Study ID Number:		Patient Initials:				Hospital Number:
MIM2_[ ] [ ] [ ]						[ ][ ][ ][ ][ ][ ][ ][ ][ ]

[ ] Legume-enriched

Day (if inpatient, tick & complete)		Day 8 [ ]		Day 9 [ ]		Day 10 [ ]		Day 11 [ ]		Day 12 [ ]		Day 13 [ ]		Day 14 [ ]		Day 15 [ ]	
Date		DD/MM/YYYY		DD/MM/YYYY		DD/MM/YYYY		DD/MM/YYYY		DD/MM/YYYY		DD/MM/YYYY		DD/MM/YYYY		DD/MM/YYYY	
Treatment Phase		☐Stabilise ☐Rehab		☐Stabilise ☐Rehab		☐Stabilise ☐Rehab		☐Stabilise ☐Rehab		☐Stabilise ☐Rehab		☐Stabilise ☐Rehab		☐Stabilise ☐Rehab		☐Stabilise ☐Rehab	
Weight (kg)		[kg][kg].[kg]		[kg][kg].[kg]		[kg][kg].[kg]		[kg][kg].[kg]		[kg][kg].[kg]		[kg][kg].[kg]		[kg][kg].[kg]		[kg][kg].[kg]	
Target ml or g per feed																	
If legume-enriched feed, target additional water per feed (ml)																	
Time of feed/extra water and amount taken (mls/g)		Feed	Water	Feed	Water	Feed	Water	Feed	Water	Feed	Water	Feed	Water	Feed	Water	Feed	Water
	1: HH:MM																
	2: HH:MM																
	3: HH:MM																
	4: HH:MM																
	5: HH:MM																
	6: HH:MM																
Total ml or g taken																	
Mark (x) if child vomited after feed		1 2 3 4 5 6		1 2 3 4 5 6		1 2 3 4 5 6		1 2 3 4 5 6		1 2 3 4 5 6		1 2 3 4 5 6		1 2 3 4 5 6		1 2 3 4 5 6	
If legume-enriched feed, record the batch number		1 2 3 4 5 6		1 2 3 4 5 6		1 2 3 4 5 6		1 2 3 4 5 6		1 2 3 4 5 6		1 2 3 4 5 6		1 2 3 4 5 6		1 2 3 4 5 6	
Any feed missed? Use codes in footnote to record reason		☐ Yes / ☐ No ____________		☐ Yes / ☐ No ____________		☐ Yes / ☐ No ____________		☐ Yes / ☐ No ____________		☐ Yes / ☐ No ____________		☐ Yes / ☐ No ____________		☐ Yes / ☐ No ____________		☐ Yes / ☐ No ____________	
Receiving feed via NG?		☐ Yes / ☐ No		☐ Yes / ☐ No		☐ Yes / ☐ No		☐ Yes / ☐ No		☐ Yes / ☐ No		☐ Yes / ☐ No		☐ Yes / ☐ No		☐ Yes / ☐ No	
Completed by:																	
Initials:																	

Footnote: 1 – Unconscious; 2 – Not present on ward at time of feed; 3 – Refused/Dislikes; 4 – Nausea or Vomiting; 5 – Other Reason (specify):________________

Case Report Form 10: Feed provision

04/06/2018

[ ] Control

Study ID Number:	Patient Initials:	Hospital Number:
MIM2_[ ][ ][ ]		[ ][ ][ ][ ][ ][ ][ ][ ][ ]

[ ] Legume-enriched

Day (if inpatient, tick & complete)		Other [ ][ ]		Other [ ][ ]		Other [ ][ ]		Other [ ][ ]		Other [ ][ ]		Other [ ][ ]		Other [ ][ ]		Other [ ][ ]	
Date		DD/MM/YYYY		DD/MM/YYYY		DD/MM/YYYY		DD/MM/YYYY		DD/MM/YYYY		DD/MM/YYYY		DD/MM/YYYY		DD/MM/YYYY	
Treatment Phase		☐Stabilise ☐Rehab		☐Stabilise ☐Rehab		☐Stabilise ☐Rehab		☐Stabilise ☐Rehab		☐Stabilise ☐Rehab		☐Stabilise ☐Rehab		☐Stabilise ☐Rehab		☐Stabilise ☐Rehab	
Weight (kg)		[kg][kg].[kg]		[kg][kg].[kg]		[kg][kg].[kg]		[kg][kg].[kg]		[kg][kg].[kg]		[kg][kg].[kg]		[kg][kg].[kg]		[kg][kg].[kg]	
Target ml or g per feed																	
If legume-enriched feed, target additional water per feed (ml)																	
Time of feed/extra water and amount taken (mls/g)		Feed	Water	Feed	Water	Feed	Water	Feed	Water	Feed	Water	Feed	Water	Feed	Water	Feed	Water
	1: HH:MM																
	2: HH:MM																
	3: HH:MM																
	4: HH:MM																
	5: HH:MM																
	6: HH:MM																
Total ml or g taken																	
Mark (x) if child vomited after feed		1 2 3 4 5 6		1 2 3 4 5 6		1 2 3 4 5 6		1 2 3 4 5 6		1 2 3 4 5 6		1 2 3 4 5 6		1 2 3 4 5 6		1 2 3 4 5 6	
If legume-enriched feed, record the batch number		1 2 3 4 5 6		1 2 3 4 5 6		1 2 3 4 5 6		1 2 3 4 5 6		1 2 3 4 5 6		1 2 3 4 5 6		1 2 3 4 5 6		1 2 3 4 5 6	
Any feed missed? Use codes in footnote to record reason		☐ Yes / ☐ No ____		☐ Yes / ☐ No ____		☐ Yes / ☐ No ____		☐ Yes / ☐ No ____		☐ Yes / ☐ No ____		☐ Yes / ☐ No ____		☐ Yes / ☐ No ____		☐ Yes / ☐ No ____	
Receiving feed via NG?		☐ Yes / ☐ No		☐ Yes / ☐ No		☐ Yes / ☐ No		☐ Yes / ☐ No		☐ Yes / ☐ No		☐ Yes / ☐ No		☐ Yes / ☐ No		☐ Yes / ☐ No	
Completed by:																	
Initials:																	

Footnote: 1 – Unconscious; 2 – Not present on ward at time of feed; 3 – Refused/Dislikes; 4 – Nausea or Vomiting; 5 – Other Reason (specify):________________

Study ID Number:	Patient Initials:	Hospital Number:
MIM2_[ ] [ ] [ ]		[ ][ ][ ][ ][ ][ ][ ][ ][ ]

Day	Adm [ ]	Day 1 [ ]	Day 2 [ ]	Day 3 [ ]	Day 4 [ ]	Day 5 [ ]	Day 6 [ ]
Date	DD/MM/YYYY	DD/MM/YYYY	DD/MM/YYYY	DD/MM/YYYY	DD/MM/YYYY	DD/MM/YYYY	DD/MM/YYYY
Weight (kg)	[kg][kg].[kg]	[kg][kg].[kg]	[kg][kg].[kg]	[kg][kg].[kg]	[kg][kg].[kg]	[kg][kg].[kg]	[kg][kg].[kg]
MUAC (cm)<br>(every third day)	[cm][cm].[cm]	[cm][cm].[cm]	[cm][cm].[cm]	[cm][cm].[cm]	[cm][cm].[cm]	[cm][cm].[cm]	[cm][cm].[cm]
Appetite Test	[ ] Pass [ ] Fail	[ ] Pass [ ] Fail	[ ] Pass [ ] Fail	[ ] Pass [ ] Fail	[ ] Pass [ ] Fail	[ ] Pass [ ] Fail	[ ] Pass [ ] Fail
Oedema score	[ ] None<br>[ ] Pre-tibial<br>[ ] Arms & legs<br>[ ] Generalised	[ ] None<br>[ ] Pre-tibial<br>[ ] Arms & legs<br>[ ] Generalised	[ ] None<br>[ ] Pre-tibial<br>[ ] Arms & legs<br>[ ] Generalised	[ ] None<br>[ ] Pre-tibial<br>[ ] Arms & legs<br>[ ] Generalised	[ ] None<br>[ ] Pre-tibial<br>[ ] Arms & legs<br>[ ] Generalised	[ ] None<br>[ ] Pre-tibial<br>[ ] Arms & legs<br>[ ] Generalised	[ ] None<br>[ ] Pre-tibial<br>[ ] Arms & legs<br>[ ] Generalised
Blood glucose (mmol/l)<br>(During stabilisation, or when indicated)							

(twice daily in stabilisation only)	HH:MM	HH:MM	HH:MM	HH:MM	HH:MM	HH:MM	HH:MM	HH:MM	HH:MM	HH:MM	HH:MM	HH:MM	HH:MM	HH:MM
Pulse rate (bpm)														
Respiratory rate (brpm)														
Oxygen sats (%)														
Ax. Temp (°C)														

Stool *(Bristol Stool Chart consistency)*																												

A: Alert; V: Responsive to voice; P: Responsive to pain; U: Unresponsive

Study ID Number:	Patient Initials:	Hospital Number:
MIM2_[ ] [ ] [ ]		[ ][ ][ ][ ][ ][ ][ ][ ][ ]

In the LAST 24 hours: any of the following reported:

Day	Adm [ ]	Day 1 [ ]	Day 2 [ ]	Day 3 [ ]	Day 4 [ ]	Day 5 [ ]	Day 6 [ ]
Date	DD/MM/YYYY	DD/MM/YYYY	DD/MM/YYYY	DD/MM/YYYY	DD/MM/YYYY	DD/MM/YYYY	DD/MM/YYYY
Vomiting<br>If yes give number:	Yes [ ] / No [ ]<br>Number	Yes [ ] / No [ ]<br>Number	Yes [ ] / No [ ]<br>Number	Yes [ ] / No [ ]<br>Number	Yes [ ] / No [ ]<br>Number	Yes [ ] / No [ ]<br>Number	Yes [ ] / No [ ]<br>Number
Abdominal pain	**Y N**	**Y N**	**Y N**	**Y N**	**Y N**	**Y N**	**Y N**
Chest in-drawing	**Y N**	**Y N**	**Y N**	**Y N**	**Y N**	**Y N**	**Y N**
Required oxygen	**Y N**	**Y N**	**Y N**	**Y N**	**Y N**	**Y N**	**Y N**
Change in consciousness	**A V P U**	**A V P U**	**A V P U**	**A V P U**	**A V P U**	**A V P U**	**A V P U**
Convulsions	**Y N**	**Y N**	**Y N**	**Y N**	**Y N**	**Y N**	**Y N**
Allergic reaction of any grade	**Y N**	**Y N**	**Y N**	**Y N**	**Y N**	**Y N**	**Y N**
Any breastfeeding	**Y N**	**Y N**	**Y N**	**Y N**	**Y N**	**Y N**	**Y N**
ReSoMal in last 24h	**Y N**	**Y N**	**Y N**	**Y N**	**Y N**	**Y N**	**Y N**
IV fluids given in last 24h	**Y N**	**Y N**	**Y N**	**Y N**	**Y N**	**Y N**	**Y N**
Currently on antibiotics?	**Y N**	**Y N**	**Y N**	**Y N**	**Y N**	**Y N**	**Y N**
If yes, specify	☐ Gentamicin<br>☐ Ceftriaxone<br>☐ Cloxacillin<br>☐ Ampiclox<br>☐Ampicillin<br>☐ Benzylpenicillin<br>☐ Metronidazole<br>☐ Chloramphenicol<br>☐ Other__________	☐ Gentamicin<br>☐ Ceftriaxone<br>☐ Cloxacillin<br>☐ Ampiclox<br>☐Ampicillin<br>☐ Benzylpenicillin<br>☐ Metronidazole<br>☐ Chloramphenicol<br>☐ Other__________	☐ Gentamicin<br>☐ Ceftriaxone<br>☐ Cloxacillin<br>☐ Ampiclox<br>☐Ampicillin<br>☐ Benzylpenicillin<br>☐ Metronidazole<br>☐ Chloramphenicol<br>☐ Other__________	☐ Gentamicin<br>☐ Ceftriaxone<br>☐ Cloxacillin<br>☐ Ampiclox<br>☐Ampicillin<br>☐ Benzylpenicillin<br>☐ Metronidazole<br>☐ Chloramphenicol<br>☐ Other__________	☐ Gentamicin<br>☐ Ceftriaxone<br>☐ Cloxacillin<br>☐ Ampiclox<br>☐Ampicillin<br>☐ Benzylpenicillin<br>☐ Metronidazole<br>☐ Chloramphenicol<br>☐ Other__________	☐ Gentamicin<br>☐ Ceftriaxone<br>☐ Cloxacillin<br>☐ Ampiclox<br>☐Ampicillin<br>☐ Benzylpenicillin<br>☐ Metronidazole<br>☐ Chloramphenicol<br>☐ Other__________	☐ Gentamicin<br>☐ Ceftriaxone<br>☐ Cloxacillin<br>☐ Ampiclox<br>☐Ampicillin<br>☐ Benzylpenicillin<br>☐ Metronidazole<br>☐ Chloramphenicol<br>☐ Other__________
Anti-TB treatment currently?	**Y N**	**Y N**	**Y N**	**Y N**	**Y N**	**Y N**	**Y N**
**ASSESSMENT COMPLETED BY:**							
**Initial**							

A: Alert; V: Responsive to voice; P: Responsive to pain; U: Unresponsive

Study ID Number:	Patient Initials:	Hospital Number:
MIM2_[ ] [ ] [ ]		[ ][ ][ ][ ][ ][ ][ ][ ][ ]

Day	Day 7 [ ]	Day 8 [ ]	Day 9 [ ]	Day 10 [ ]	Day 11 [ ]	Day 12 [ ]	Day 13 [ ]
Date	DD/MM/YYYY	DD/MM/YYYY	DD/MM/YYYY	DD/MM/YYYY	DD/MM/YYYY	DD/MM/YYYY	DD/MM/YYYY
Weight (kg)	[kg][kg].[kg]	[kg][kg].[kg]	[kg][kg].[kg]	[kg][kg].[kg]	[kg][kg].[kg]	[kg][kg].[kg]	[kg][kg].[kg]
MUAC (cm)<br>(every third day)	[cm][cm].[cm]	[cm][cm].[cm]	[cm][cm].[cm]	[cm][cm].[cm]	[cm][cm].[cm]	[cm][cm].[cm]	[cm][cm].[cm]
Appetite Test	[ ] Pass [ ] Fail	[ ] Pass [ ] Fail	[ ] Pass [ ] Fail	[ ] Pass [ ] Fail	[ ] Pass [ ] Fail	[ ] Pass [ ] Fail	[ ] Pass [ ] Fail
Oedema score	[ ] None<br>[ ] Pre-tibial<br>[ ] Arms & legs<br>[ ] Generalised	[ ] None<br>[ ] Pre-tibial<br>[ ] Arms & legs<br>[ ] Generalised	[ ] None<br>[ ] Pre-tibial<br>[ ] Arms & legs<br>[ ] Generalised	[ ] None<br>[ ] Pre-tibial<br>[ ] Arms & legs<br>[ ] Generalised	[ ] None<br>[ ] Pre-tibial<br>[ ] Arms & legs<br>[ ] Generalised	[ ] None<br>[ ] Pre-tibial<br>[ ] Arms & legs<br>[ ] Generalised	[ ] None<br>[ ] Pre-tibial<br>[ ] Arms & legs<br>[ ] Generalised
Blood glucose (mmol/l)<br>(During stabilisation, or when indicated)							

(twice daily in stabilisation only)	HH:MM	HH:MM	HH:MM	HH:MM	HH:MM	HH:MM	HH:MM	HH:MM	HH:MM	HH:MM	HH:MM	HH:MM	HH:MM	HH:MM
Pulse rate (bpm)														
Respiratory rate (brpm)														
Oxygen sats (%)														
Ax. Temp (°C)														

Stool *(Bristol Stool Chart consistency)*							

A: Alert; V: Responsive to voice; P: Responsive to pain; U: Unresponsive

Study ID Number:		Patient Initials:				Hospital Number:
MIM2_[ ] [ ] [ ]						[ ][ ][ ][ ][ ][ ][ ][ ][ ]

In the LAST 24 hours: any of the following reported:							
Day	Day 7 [ ]	Day 8 [ ]	Day 9 [ ]	Day 10 [ ]	Day 11 [ ]	Day 12 [ ]	Day 13 [ ]
Date	DD/MM/YYYY	DD/MM/YYYY	DD/MM/YYYY	DD/MM/YYYY	DD/MM/YYYY	DD/MM/YYYY	DD/MM/YYYY
Vomiting<br>If yes give number	Yes [ ] / No [ ]<br>Number	Yes [ ] / No [ ]<br>Number	Yes [ ] / No [ ]<br>Number	Yes [ ] / No [ ]<br>Number	Yes [ ] / No [ ]<br>Number	Yes [ ] / No [ ]<br>Number	Yes [ ] / No [ ]<br>Number
Abdominal pain	**Y N**	**Y N**	**Y N**	**Y N**	**Y N**	**Y N**	**Y N**
Chest in-drawing	**Y N**	**Y N**	**Y N**	**Y N**	**Y N**	**Y N**	**Y N**
Required oxygen	**Y N**	**Y N**	**Y N**	**Y N**	**Y N**	**Y N**	**Y N**
Change in consciousness	**A V P U**	**A V P U**	**A V P U**	**A V P U**	**A V P U**	**A V P U**	**A V P U**
Convulsions	**Y N**	**Y N**	**Y N**	**Y N**	**Y N**	**Y N**	**Y N**
Allergic reaction of any grade	**Y N**	**Y N**	**Y N**	**Y N**	**Y N**	**Y N**	**Y N**
Any breastfeeding	**Y N**	**Y N**	**Y N**	**Y N**	**Y N**	**Y N**	**Y N**
ReSoMal in last 24h	**Y N**	**Y N**	**Y N**	**Y N**	**Y N**	**Y N**	**Y N**
IV fluids given in last 24h	**Y N**	**Y N**	**Y N**	**Y N**	**Y N**	**Y N**	**Y N**
Currently on antibiotics?	**Y N**	**Y N**	**Y N**	**Y N**	**Y N**	**Y N**	**Y N**
If yes, specify	☐ Gentamicin<br>☐ Ceftriaxone<br>☐ Cloxacillin<br>☐ Ampiclox<br>☐Ampicillin<br>☐ Benzylpenicillin<br>☐ Metronidazole<br>☐ Chloramphenicol<br>☐ Other__________	☐ Gentamicin<br>☐ Ceftriaxone<br>☐ Cloxacillin<br>☐ Ampiclox<br>☐Ampicillin<br>☐ Benzylpenicillin<br>☐ Metronidazole<br>☐ Chloramphenicol<br>☐ Other__________	☐ Gentamicin<br>☐ Ceftriaxone<br>☐ Cloxacillin<br>☐ Ampiclox<br>☐Ampicillin<br>☐ Benzylpenicillin<br>☐ Metronidazole<br>☐ Chloramphenicol<br>☐ Other__________	☐ Gentamicin<br>☐ Ceftriaxone<br>☐ Cloxacillin<br>☐ Ampiclox<br>☐Ampicillin<br>☐ Benzylpenicillin<br>☐ Metronidazole<br>☐ Chloramphenicol<br>☐ Other__________	☐ Gentamicin<br>☐ Ceftriaxone<br>☐ Cloxacillin<br>☐ Ampiclox<br>☐Ampicillin<br>☐ Benzylpenicillin<br>☐ Metronidazole<br>☐ Chloramphenicol<br>☐ Other__________	☐ Gentamicin<br>☐ Ceftriaxone<br>☐ Cloxacillin<br>☐ Ampiclox<br>☐Ampicillin<br>☐ Benzylpenicillin<br>☐ Metronidazole<br>☐ Chloramphenicol<br>☐ Other__________	☐ Gentamicin<br>☐ Ceftriaxone<br>☐ Cloxacillin<br>☐ Ampiclox<br>☐Ampicillin<br>☐ Benzylpenicillin<br>☐ Metronidazole<br>☐ Chloramphenicol<br>☐ Other__________
Anti-TB treatment currently?	**Y N**	**Y N**	**Y N**	**Y N**	**Y N**	**Y N**	**Y N**
**ASSESSMENT COMPLETED BY:**							
**Initial**							

A: Alert; V: Responsive to voice; P: Responsive to pain; U: Unresponsive

Study ID Number:	Patient Initials:			Hospital Number:
MIM2_[ ] [ ] [ ]				[ ][ ][ ][ ][ ][ ][ ][ ][ ]

Day	Day 14 [ ]	Other day [ ][ ]	Other day [ ][ ]	Other day [ ][ ]	Other day [ ][ ]	Other day [ ][ ]	Other day [ ][ ]
Date	DD/MM/YYYY	DD/MM/YYYY	DD/MM/YYYY	DD/MM/YYYY	DD/MM/YYYY	DD/MM/YYYY	DD/MM/YYYY
Weight (kg)	[kg][kg].[kg]	[kg][kg].[kg]	[kg][kg].[kg]	[kg][kg].[kg]	[kg][kg].[kg]	[kg][kg].[kg]	[kg][kg].[kg]
MUAC (cm) (every third day)	[cm][cm].[cm]	[cm][cm].[cm]	[cm][cm].[cm]	[cm][cm].[cm]	[cm][cm].[cm]	[cm][cm].[cm]	[cm][cm].[cm]
Appetite Test	[ ] Pass [ ] Fail	[ ] Pass [ ] Fail	[ ] Pass [ ] Fail	[ ] Pass [ ] Fail	[ ] Pass [ ] Fail	[ ] Pass [ ] Fail	[ ] Pass [ ] Fail
Oedema score	[ ] None<br>[ ] Pre-tibial<br>[ ] Arms & legs<br>[ ] Generalised	[ ] None<br>[ ] Pre-tibial<br>[ ] Arms & legs<br>[ ] Generalised	[ ] None<br>[ ] Pre-tibial<br>[ ] Arms & legs<br>[ ] Generalised	[ ] None<br>[ ] Pre-tibial<br>[ ] Arms & legs<br>[ ] Generalised	[ ] None<br>[ ] Pre-tibial<br>[ ] Arms & legs<br>[ ] Generalised	[ ] None<br>[ ] Pre-tibial<br>[ ] Arms & legs<br>[ ] Generalised	[ ] None<br>[ ] Pre-tibial<br>[ ] Arms & legs<br>[ ] Generalised
Blood glucose (mmol/l) (During stabilisation, or when indicated)							

(twice daily in stabilisation only)	HH:MM	HH:MM	HH:MM	HH:MM	HH:MM	HH:MM	HH:MM	HH:MM	HH:MM	HH:MM	HH:MM	HH:MM	HH:MM	HH:MM
Pulse rate (bpm)														
Respiratory rate (brpm)														
Oxygen sats (%)														
Ax. Temp (°C)														

Stool *(Bristol Stool Chart consistency)*																												

A: Alert; V: Responsive to voice; P: Responsive to pain; U: Unresponsive

Study ID Number:	Patient Initials:	Hospital Number:
MIM2_[ ] [ ] [ ]		[ ][ ][ ][ ][ ][ ][ ][ ][ ]

In the LAST 24 hours: any of the following reported:							
Day	Day 14 [ ]	Other day [ ][ ]	Other day [ ][ ]	Other day [ ][ ]	Other day [ ][ ]	Other day [ ][ ]	Other day [ ][ ]
Date	DD/MM/YYYY	DD/MM/YYYY	DD/MM/YYYY	DD/MM/YYYY	DD/MM/YYYY	DD/MM/YYYY	DD/MM/YYYY
Vomiting<br>If yes give number	Yes [ ] / No [ ]<br>Number	Yes [ ] / No [ ]<br>Number	Yes [ ] / No [ ]<br>Number	Yes [ ] / No [ ]<br>Number	Yes [ ] / No [ ]<br>Number	Yes [ ] / No [ ]<br>Number	Yes [ ] / No [ ]<br>Number
Abdominal pain	**Y N**	**Y N**	**Y N**	**Y N**	**Y N**	**Y N**	**Y N**
Chest in-drawing	**Y N**	**Y N**	**Y N**	**Y N**	**Y N**	**Y N**	**Y N**
Required oxygen	**Y N**	**Y N**	**Y N**	**Y N**	**Y N**	**Y N**	**Y N**
Change in consciousness	**A V P U**	**A V P U**	**A V P U**	**A V P U**	**A V P U**	**A V P U**	**A V P U**
Convulsions	**Y N**	**Y N**	**Y N**	**Y N**	**Y N**	**Y N**	**Y N**
Allergic reaction of any grade	**Y N**	**Y N**	**Y N**	**Y N**	**Y N**	**Y N**	**Y N**
Any breastfeeding	**Y N**	**Y N**	**Y N**	**Y N**	**Y N**	**Y N**	**Y N**
ReSoMal in last 24h	**Y N**	**Y N**	**Y N**	**Y N**	**Y N**	**Y N**	**Y N**
IV fluids given in last 24h	**Y N**	**Y N**	**Y N**	**Y N**	**Y N**	**Y N**	**Y N**
Currently on antibiotics?	**Y N**	**Y N**	**Y N**	**Y N**	**Y N**	**Y N**	**Y N**
If yes, specify	☐ Gentamicin<br>☐ Ceftriaxone<br>☐ Cloxacillin<br>☐ Ampiclox<br>☐Ampicillin<br>☐ Benzylpenicillin<br>☐ Metronidazole<br>☐ Chloramphenicol<br>☐ Other__________	☐ Gentamicin<br>☐ Ceftriaxone<br>☐ Cloxacillin<br>☐ Ampiclox<br>☐Ampicillin<br>☐ Benzylpenicillin<br>☐ Metronidazole<br>☐ Chloramphenicol<br>☐ Other__________	☐ Gentamicin<br>☐ Ceftriaxone<br>☐ Cloxacillin<br>☐ Ampiclox<br>☐Ampicillin<br>☐ Benzylpenicillin<br>☐ Metronidazole<br>☐ Chloramphenicol<br>☐ Other__________	☐ Gentamicin<br>☐ Ceftriaxone<br>☐ Cloxacillin<br>☐ Ampiclox<br>☐Ampicillin<br>☐ Benzylpenicillin<br>☐ Metronidazole<br>☐ Chloramphenicol<br>☐ Other__________	☐ Gentamicin<br>☐ Ceftriaxone<br>☐ Cloxacillin<br>☐ Ampiclox<br>☐Ampicillin<br>☐ Benzylpenicillin<br>☐ Metronidazole<br>☐ Chloramphenicol<br>☐ Other__________	☐ Gentamicin<br>☐ Ceftriaxone<br>☐ Cloxacillin<br>☐ Ampiclox<br>☐Ampicillin<br>☐ Benzylpenicillin<br>☐ Metronidazole<br>☐ Chloramphenicol<br>☐ Other__________	☐ Gentamicin<br>☐ Ceftriaxone<br>☐ Cloxacillin<br>☐ Ampiclox<br>☐Ampicillin<br>☐ Benzylpenicillin<br>☐ Metronidazole<br>☐ Chloramphenicol<br>☐ Other__________
Anti-TB treatment currently?	**Y N**	**Y N**	**Y N**	**Y N**	**Y N**	**Y N**	**Y N**
**ASSESSMENT COMPLETED BY:**							
**Initial**							

A: Alert; V: Responsive to voice; P: Responsive to pain; U: Unresponsive

Study ID Number:	Patient Initials:	Hospital Number:
MIM2_[ ][ ][ ]		[ ][ ][ ][ ][ ][ ][ ][ ][ ]

Day of Admission		Admission	Day 1	Day 2	Day 3	Day 4	Day 5	Day 6	Day 7
Weight (g) of legume-enriched product per feed		[ ][ ][ ]g	[ ][ ][ ]g	[ ][ ][ ]g	[ ][ ][ ]g	[ ][ ][ ]g	[ ][ ][ ]g	[ ][ ][ ]g	[ ][ ][ ]g
Mineral Mix	Prescribed daily volume (ml)	[ ][ ].[ ]ml	[ ][ ].[ ]ml	[ ][ ].[ ]ml	[ ][ ].[ ]ml	[ ][ ].[ ]ml	[ ][ ].[ ]ml	[ ][ ].[ ]ml	[ ][ ].[ ]ml
	Date of prescription	DD/MM/YYYY	DD/MM/YYYY	DD/MM/YYYY	DD/MM/YYYY	DD/MM/YYYY	DD/MM/YYYY	DD/MM/YYYY	DD/MM/YYYY
	Clinician Initials	[ ][ ][ ]	[ ][ ][ ]	[ ][ ][ ]	[ ][ ][ ]	[ ][ ][ ]	[ ][ ][ ]	[ ][ ][ ]	[ ][ ][ ]
	Volume (ml) administered	[ ][ ].[ ]ml	[ ][ ].[ ]ml	[ ][ ].[ ]ml	[ ][ ].[ ]ml	[ ][ ].[ ]ml	[ ][ ].[ ]ml	[ ][ ].[ ]ml	[ ][ ].[ ]ml
	Date of administration	DD/MM/YYYY	DD/MM/YYYY	DD/MM/YYYY	DD/MM/YYYY	DD/MM/YYYY	DD/MM/YYYY	DD/MM/YYYY	DD/MM/YYYY
	Time (24hr) of administration	HH:MM	HH:MM	HH:MM	HH:MM	HH:MM	HH:MM	HH:MM	HH:MM
	Nurse Initials	[ ][ ][ ]	[ ][ ][ ]	[ ][ ][ ]	[ ][ ][ ]	[ ][ ][ ]	[ ][ ][ ]	[ ][ ][ ]	[ ][ ][ ]
Grovit	Prescribed daily volume (ml)	[ ][ ].[ ]ml	[ ][ ].[ ]ml	[ ][ ].[ ]ml	[ ][ ].[ ]ml	[ ][ ].[ ]ml	[ ][ ].[ ]ml	[ ][ ].[ ]ml	[ ][ ].[ ]ml
	Date of prescription	DD/MM/YYYY	DD/MM/YYYY	DD/MM/YYYY	DD/MM/YYYY	DD/MM/YYYY	DD/MM/YYYY	DD/MM/YYYY	DD/MM/YYYY
	Clinician Initials	[ ][ ][ ]	[ ][ ][ ]	[ ][ ][ ]	[ ][ ][ ]	[ ][ ][ ]	[ ][ ][ ]	[ ][ ][ ]	[ ][ ][ ]
	Volume (ml) administered	[ ][ ].[ ]ml	[ ][ ].[ ]ml	[ ][ ].[ ]ml	[ ][ ].[ ]ml	[ ][ ].[ ]ml	[ ][ ].[ ]ml	[ ][ ].[ ]ml	[ ][ ].[ ]ml
	Date of administration	DD/MM/YYYY	DD/MM/YYYY	DD/MM/YYYY	DD/MM/YYYY	DD/MM/YYYY	DD/MM/YYYY	DD/MM/YYYY	DD/MM/YYYY
	Time (24hr) of administration	HH:MM	HH:MM	HH:MM	HH:MM	HH:MM	HH:MM	HH:MM	HH:MM
	Nurse Initials	[ ][ ][ ]	[ ][ ][ ]	[ ][ ][ ]	[ ][ ][ ]	[ ][ ][ ]	[ ][ ][ ]	[ ][ ][ ]	[ ][ ][ ]
Zinc Sulphate	Prescribed daily volume (ml)	[ ][ ].[ ]ml	[ ][ ].[ ]ml	[ ][ ].[ ]ml	[ ][ ].[ ]ml	[ ][ ].[ ]ml	[ ][ ].[ ]ml	[ ][ ].[ ]ml	[ ][ ].[ ]ml
	Date of prescription	DD/MM/YYYY	DD/MM/YYYY	DD/MM/YYYY	DD/MM/YYYY	DD/MM/YYYY	DD/MM/YYYY	DD/MM/YYYY	DD/MM/YYYY
	Clinician Initials	[ ][ ][ ]	[ ][ ][ ]	[ ][ ][ ]	[ ][ ][ ]	[ ][ ][ ]	[ ][ ][ ]	[ ][ ][ ]	[ ][ ][ ]
	Volume (ml) administered	[ ][ ].[ ]ml	[ ][ ].[ ]ml	[ ][ ].[ ]ml	[ ][ ].[ ]ml	[ ][ ].[ ]ml	[ ][ ].[ ]ml	[ ][ ].[ ]ml	[ ][ ].[ ]ml
	Date of administration	DD/MM/YYYY	DD/MM/YYYY	DD/MM/YYYY	DD/MM/YYYY	DD/MM/YYYY	DD/MM/YYYY	DD/MM/YYYY	DD/MM/YYYY
	Time (24hr) of administration	HH:MM	HH:MM	HH:MM	HH:MM	HH:MM	HH:MM	HH:MM	HH:MM
	Nurse Initials	[ ][ ][ ]	[ ][ ][ ]	[ ][ ][ ]	[ ][ ][ ]	[ ][ ][ ]	[ ][ ][ ]	[ ][ ][ ]	[ ][ ][ ]

Study ID Number:	Patient Initials:	Hospital Number:
MIM2_[ ][ ][ ]		[ ][ ][ ][ ][ ][ ][ ][ ][ ][ ]

Day of Admission		Day 8	Day 9	Day 10	Day 11	Day 12	Day 13	Day 14	Day 15
Weight (g) of legume-enriched product per feed		[ ][ ][ ]g	[ ][ ][ ]g	[ ][ ][ ]g	[ ][ ][ ]g	[ ][ ][ ]g	[ ][ ][ ]g	[ ][ ][ ]g	[ ][ ][ ]g
Mineral Mix	Prescribed daily volume (ml)	[ ][ ].[ ]ml	[ ][ ].[ ]ml	[ ][ ].[ ]ml	[ ][ ].[ ]ml	[ ][ ].[ ]ml	[ ][ ].[ ]ml	[ ][ ].[ ]ml	[ ][ ].[ ]ml
	Date of prescription	DD/MM/YYYY	DD/MM/YYYY	DD/MM/YYYY	DD/MM/YYYY	DD/MM/YYYY	DD/MM/YYYY	DD/MM/YYYY	DD/MM/YYYY
	Clinician Initials	[ ][ ][ ]	[ ][ ][ ]	[ ][ ][ ]	[ ][ ][ ]	[ ][ ][ ]	[ ][ ][ ]	[ ][ ][ ]	[ ][ ][ ]
	Volume (ml) administered	[ ][ ].[ ]ml	[ ][ ].[ ]ml	[ ][ ].[ ]ml	[ ][ ].[ ]ml	[ ][ ].[ ]ml	[ ][ ].[ ]ml	[ ][ ].[ ]ml	[ ][ ].[ ]ml
	Date of administration	DD/MM/YYYY	DD/MM/YYYY	DD/MM/YYYY	DD/MM/YYYY	DD/MM/YYYY	DD/MM/YYYY	DD/MM/YYYY	DD/MM/YYYY
	Time (24hr) of administration	HH:MM	HH:MM	HH:MM	HH:MM	HH:MM	HH:MM	HH:MM	HH:MM
	Nurse Initials	[ ][ ][ ]	[ ][ ][ ]	[ ][ ][ ]	[ ][ ][ ]	[ ][ ][ ]	[ ][ ][ ]	[ ][ ][ ]	[ ][ ][ ]
Grovit	Prescribed daily volume (ml)	[ ][ ].[ ]ml	[ ][ ].[ ]ml	[ ][ ].[ ]ml	[ ][ ].[ ]ml	[ ][ ].[ ]ml	[ ][ ].[ ]ml	[ ][ ].[ ]ml	[ ][ ].[ ]ml
	Date of prescription	DD/MM/YYYY	DD/MM/YYYY	DD/MM/YYYY	DD/MM/YYYY	DD/MM/YYYY	DD/MM/YYYY	DD/MM/YYYY	DD/MM/YYYY
	Clinician Initials	[ ][ ][ ]	[ ][ ][ ]	[ ][ ][ ]	[ ][ ][ ]	[ ][ ][ ]	[ ][ ][ ]	[ ][ ][ ]	[ ][ ][ ]
	Volume (ml) administered	[ ][ ].[ ]ml	[ ][ ].[ ]ml	[ ][ ].[ ]ml	[ ][ ].[ ]ml	[ ][ ].[ ]ml	[ ][ ].[ ]ml	[ ][ ].[ ]ml	[ ][ ].[ ]ml
	Date of administration	DD/MM/YYYY	DD/MM/YYYY	DD/MM/YYYY	DD/MM/YYYY	DD/MM/YYYY	DD/MM/YYYY	DD/MM/YYYY	DD/MM/YYYY
	Time (24hr) of administration	HH:MM	HH:MM	HH:MM	HH:MM	HH:MM	HH:MM	HH:MM	HH:MM
	Nurse Initials	[ ][ ][ ]	[ ][ ][ ]	[ ][ ][ ]	[ ][ ][ ]	[ ][ ][ ]	[ ][ ][ ]	[ ][ ][ ]	[ ][ ][ ]
Zinc Sulphate	Prescribed daily volume (ml)	[ ][ ].[ ]ml	[ ][ ].[ ]ml	[ ][ ].[ ]ml	[ ][ ].[ ]ml	[ ][ ].[ ]ml	[ ][ ].[ ]ml	[ ][ ].[ ]ml	[ ][ ].[ ]ml
	Date of prescription	DD/MM/YYYY	DD/MM/YYYY	DD/MM/YYYY	DD/MM/YYYY	DD/MM/YYYY	DD/MM/YYYY	DD/MM/YYYY	DD/MM/YYYY
	Clinician Initials	[ ][ ][ ]	[ ][ ][ ]	[ ][ ][ ]	[ ][ ][ ]	[ ][ ][ ]	[ ][ ][ ]	[ ][ ][ ]	[ ][ ][ ]
	Volume (ml) administered	[ ][ ].[ ]ml	[ ][ ].[ ]ml	[ ][ ].[ ]ml	[ ][ ].[ ]ml	[ ][ ].[ ]ml	[ ][ ].[ ]ml	[ ][ ].[ ]ml	[ ][ ].[ ]ml
	Date of administration	DD/MM/YYYY	DD/MM/YYYY	DD/MM/YYYY	DD/MM/YYYY	DD/MM/YYYY	DD/MM/YYYY	DD/MM/YYYY	DD/MM/YYYY
	Time (24hr) of administration	HH:MM	HH:MM	HH:MM	HH:MM	HH:MM	HH:MM	HH:MM	HH:MM
	Nurse Initials	[ ][ ][ ]	[ ][ ][ ]	[ ][ ][ ]	[ ][ ][ ]	[ ][ ][ ]	[ ][ ][ ]	[ ][ ][ ]	[ ][ ][ ]

Study ID Number:	Patient Initials:			Hospital Number:
MIM2_ [ ] [ ] [ ]				[ ][ ][ ][ ][ ][ ][ ][ ][ ]

Day of Admission		Day [ ][ ]	Day [ ][ ]	Day [ ][ ]	Day [ ][ ]	Day [ ][ ]	Day [ ][ ]	Day [ ][ ]	Day [ ][ ]
Weight (g) of legume-enriched product per feed		[ ][ ][ ]g	[ ][ ][ ]g	[ ][ ][ ]g	[ ][ ][ ]g	[ ][ ][ ]g	[ ][ ][ ]g	[ ][ ][ ]g	[ ][ ][ ]g
Mineral Mix	Prescribed daily volume (ml)	[ ][ ].[ ]ml	[ ][ ].[ ]ml	[ ][ ].[ ]ml	[ ][ ].[ ]ml	[ ][ ].[ ]ml	[ ][ ].[ ]ml	[ ][ ].[ ]ml	[ ][ ].[ ]ml
	Date of prescription	DD/MM/YYYY	DD/MM/YYYY	DD/MM/YYYY	DD/MM/YYYY	DD/MM/YYYY	DD/MM/YYYY	DD/MM/YYYY	DD/MM/YYYY
	Clinician Initials	[ ][ ][ ]	[ ][ ][ ]	[ ][ ][ ]	[ ][ ][ ]	[ ][ ][ ]	[ ][ ][ ]	[ ][ ][ ]	[ ][ ][ ]
	Volume (ml) administered	[ ][ ].[ ]ml	[ ][ ].[ ]ml	[ ][ ].[ ]ml	[ ][ ].[ ]ml	[ ][ ].[ ]ml	[ ][ ].[ ]ml	[ ][ ].[ ]ml	[ ][ ].[ ]ml
	Date of administration	DD/MM/YYYY	DD/MM/YYYY	DD/MM/YYYY	DD/MM/YYYY	DD/MM/YYYY	DD/MM/YYYY	DD/MM/YYYY	DD/MM/YYYY
	Time (24hr) of administration	HH:MM	HH:MM	HH:MM	HH:MM	HH:MM	HH:MM	HH:MM	HH:MM
	Nurse Initials	[ ][ ][ ]	[ ][ ][ ]	[ ][ ][ ]	[ ][ ][ ]	[ ][ ][ ]	[ ][ ][ ]	[ ][ ][ ]	[ ][ ][ ]
Grovit	Prescribed daily volume (ml)	[ ][ ].[ ]ml	[ ][ ].[ ]ml	[ ][ ].[ ]ml	[ ][ ].[ ]ml	[ ][ ].[ ]ml	[ ][ ].[ ]ml	[ ][ ].[ ]ml	[ ][ ].[ ]ml
	Date of prescription	DD/MM/YYYY	DD/MM/YYYY	DD/MM/YYYY	DD/MM/YYYY	DD/MM/YYYY	DD/MM/YYYY	DD/MM/YYYY	DD/MM/YYYY
	Clinician Initials	[ ][ ][ ]	[ ][ ][ ]	[ ][ ][ ]	[ ][ ][ ]	[ ][ ][ ]	[ ][ ][ ]	[ ][ ][ ]	[ ][ ][ ]
	Volume (ml) administered	[ ][ ].[ ]ml	[ ][ ].[ ]ml	[ ][ ].[ ]ml	[ ][ ].[ ]ml	[ ][ ].[ ]ml	[ ][ ].[ ]ml	[ ][ ].[ ]ml	[ ][ ].[ ]ml
	Date of administration	DD/MM/YYYY	DD/MM/YYYY	DD/MM/YYYY	DD/MM/YYYY	DD/MM/YYYY	DD/MM/YYYY	DD/MM/YYYY	DD/MM/YYYY
	Time (24hr) of administration	HH:MM	HH:MM	HH:MM	HH:MM	HH:MM	HH:MM	HH:MM	HH:MM
	Nurse Initials	[ ][ ][ ]	[ ][ ][ ]	[ ][ ][ ]	[ ][ ][ ]	[ ][ ][ ]	[ ][ ][ ]	[ ][ ][ ]	[ ][ ][ ]
Zinc Sulphate	Prescribed daily volume (ml)	[ ][ ].[ ]ml	[ ][ ].[ ]ml	[ ][ ].[ ]ml	[ ][ ].[ ]ml	[ ][ ].[ ]ml	[ ][ ].[ ]ml	[ ][ ].[ ]ml	[ ][ ].[ ]ml
	Date of prescription	DD/MM/YYYY	DD/MM/YYYY	DD/MM/YYYY	DD/MM/YYYY	DD/MM/YYYY	DD/MM/YYYY	DD/MM/YYYY	DD/MM/YYYY
	Clinician Initials	[ ][ ][ ]	[ ][ ][ ]	[ ][ ][ ]	[ ][ ][ ]	[ ][ ][ ]	[ ][ ][ ]	[ ][ ][ ]	[ ][ ][ ]
	Volume (ml) administered	[ ][ ].[ ]ml	[ ][ ].[ ]ml	[ ][ ].[ ]ml	[ ][ ].[ ]ml	[ ][ ].[ ]ml	[ ][ ].[ ]ml	[ ][ ].[ ]ml	[ ][ ].[ ]ml
	Date of administration	DD/MM/YYYY	DD/MM/YYYY	DD/MM/YYYY	DD/MM/YYYY	DD/MM/YYYY	DD/MM/YYYY	DD/MM/YYYY	DD/MM/YYYY
	Time (24hr) of administration	HH:MM	HH:MM	HH:MM	HH:MM	HH:MM	HH:MM	HH:MM	HH:MM
	Nurse Initials	[ ][ ][ ]	[ ][ ][ ]	[ ][ ][ ]	[ ][ ][ ]	[ ][ ][ ]	[ ][ ][ ]	[ ][ ][ ]	[ ][ ][ ]
