# Supplementary material for: Modifying Intestinal Integrity and Micro Biome in Severe Malnutrition with Legume-Based Feeds (MIMBLE 2.0): protocol for a phase II refined feed and intervention trial
Source: Wellcome Open Res. 2018 Aug 2;3:95. [Version 1] doi: 10.12688/wellcomeopenres.14706.1 (PMC6171552; doi:10.12688/wellcomeopenres.14706.1)
Supplement: Peer review history

# Peer review: Referee response for version 1

**DOI:** 10.21956/wellcomeopenres.16015.r33704

**Published:** 2018-09-24

**Authors:** Paluku Bahwere

**Affiliations:** 1 Valid International, Oxford, UK

The article describes very nicely the planned study. The study addresses a topic area that needs to be revised since the profile of children currently starting treatment as an inpatient has changed since the adoption of the community based-management of acute malnutrition (CMAM). If the area of the research, the CMAM programme has a well-functioning community component, children are likely to be admitted with less body function disturbance than when the current standard diets were developed.

 In their introduction, Walsh and colleagues state that the standard formulations F75/F100  have not been able to reduce case-fatality and use this statement to partly justify the need for a new Formulation. Although case-fatality of complicated SAM remains high, there is evidence that the introduction of these standards formulations have been instrumental in obtaining the drop of case fatality from around 30 to 50% to below 10% provided that the adherence to the protocol is good. We think that this statement needs to be adjusted. However, it is correct that it has been difficult to reduce further the case-fatality of children requiring initial inpatient care and exploring the contribution of legumes floor and lactose free formula is important.

 There are also areas of the methods section that may need to be revised to provide more details. Indeed more details are needed on the nutrition profile of the experimental formula, on the cooking process of the chickpea, on the rationale of using the energy density of 2kcal/ml and on the calculation of the sample size. Regarding details on the nutrition profile, it is important to have details on macro and micronutrients profile, amino-acid profile and PDCAAS, fatty acids profile and proportion of energy from omega-3 and from omega-6, phytic acid and iron and phytic acid and zinc ratios. This information will be instrumental in convincing the nutrition community that the formulation has a suitable nutrition profile. It will also help other researchers who may want to replicate the study.

 Regarding the cooking process, it is important to provide details on how chickpea will be processed /cooked as we have recently learn that the tolerability of ready-to-use therapeutic food is not the same when the grains were roasted and when the floor was obtained by extrusion cooking. Inappropriate cooking may lead to poor tolerability with symptoms appearing after 10 to 14 days. Again, this information is critical for those who want to replicate the study.

 Regarding the chosen energy density, the current recommendation is to initiate the nutrition rehabilitation with protein and energy intake that can be tolerated by the disturbed metabolic system. High intake of protein and energy at the initiation of treatment has been associated with increased case-fatality. Although for this research you have planned to restrict the intake of the experimental formula to the recommended energy intake of treatment phase 1, transition phase and phase 2,this may not be easy when the product will be used in routine. Thus, it is important to expand on the rational of the chosen density and its safety.

 Regarding sample size, because the main benefits of the new formula is to improve gut function and reduce 90-days mortality, I could have expected that the sample size will be calculated using one or the two parameters. But because MUAC has been chosen as the better proxy of these 2 parameters, there is a need to define the expected difference or of the smallest clinically meaningful difference. The sample size should then be calculated based on this and taking into account that the planned study aims at demonstrating superiority of  the experimental formula. A calculation, indicates that the sample size of 80 SAM per arm (those reaching the 90 days time-point) is adequate to demonstrate superiority if the difference between arms is 0.5 cm. Note that most regulatory bodies (including WHO) will not consider absence of difference in a superiority trial to be considered as proof of equivalence.

 In addition to the additional details suggested above, I would like to suggest collection of some other parameters that may strengthen the evidence at acceptable extra-cost and efforts. Estimation of increment of lean mass difference may be added at a very limited cost using the simplest single-frequency bio-impedance analysis device. Indeed, plan based formulation are accused of promoting fat deposition rather than lean mass deposition. It will be important to demonstrate that this is not the case. Also, we recently published the results of study showing that reducing milk content of RUTF combined with the optimisation of phytic acid and iron molar ratio improves the correction of anaemia and iron deficiency. This finding suggests that some milk components inhibit iron absorption more than legumes anti-nutrients when iron and vitamin c contents of the diet are optimised. If similar results are observed with the experimental formula, the finding will provide an additional rationale for adopting this formula. Existing cheap laboratory tests will allow the addition of the assessment of haemoglobin and iron status at minimum increase in overall research budget.

I have read this submission. I believe that I have an appropriate level of expertise to confirm that it is of an acceptable scientific standard.

# Peer review: Referee response for version 1

**DOI:** 10.21956/wellcomeopenres.16015.r33640

**Published:** 2018-08-31

**Authors:** Thomas Preston

**Affiliations:** 1 Scottish Universities Environmental Research Centre, East Kilbride, UK; 2 University of Glasgow, Glasgow, UK

Walsh and colleagues plan an open trial to compare the benefit of a legume flour-enriched formula with a conventional milk formula in Ugandan children with SAM. The objective is important and topical.

 The proposed formula has two functions, to improve gut function while providing nutritional support. The improved formula is lactose-free, as brush border enzyme deficiency is common in SAM and undigested lactose will promote osmotic diarrhoea. However there are other components of a milk-based diet that may disrupt a damaged gut. For instance, there is increasing awareness of immune activation by b-casein sub-types. This has led to the discovery that some diagnoses of lactose intolerance in susceptible individuals may be due more correctly to immune activation by a1 b-casein. If this were widespread, then lactose-free skimmed milk powder may continue to disrupt small intestinal function through immune system activation. The study design has taken sufficient precaution to identify, record and act upon any adverse events, including any that may occur in response to the formula. The alternative may have been to develop a wholly plant-based formula. But this would have required much more preliminary work to prove it was palatable and capable of delivering adequate nutrition.

 Grain legumes, in particular chickpea flour, has been chosen as the food to supplement lactose-free skimmed milk powder. This is the vector chosen to improve gut barrier function, by virtue of its fermentable dietary fibre content.  Short chain fatty acids produced in the large bowel are pleiotropic agents with some of their benefit coming from improved barrier function.  In terms of the RATIONALE of this proposal, it is not explained how a large bowel function can impact the small bowel, i.e., where nutrients are digested and absorbed. No evidence is presented for there being significant microbial  fermentation in the terminal ileum allowing SCFA to act on local barrier function.  Alternatively, SCFA action in the large bowel could have remote effects, possibly acting through L-cell released hormones, such as glucagon like peptides. This was not discussed. However, the MIMBLE pilot study showed encouraging results.   

 The other role of the formula is to provide adequate nutrition.  In the absence of this information, a crude calculation reveals approximately one third of the protein comes from the chickpea.  The adequacy of the amino acid supply from the new formula should be calculated, at least in theory. This would match amino acid score and protein digestibility against the requirements. Methionine will be the first limiting amino acid in the legume protein, but as milk protein has an excellent score, the blend is likely to be adequate, but this should be demonstrated.  Perhaps the greater issue is that grain legumes contain anti-nutritional compounds or factors (ANFs) that may reduce the bioavailability of dietary protein (and minerals) from the combined formula, not just the legume flour. Choice of variety, its processing and preparation can improve the situation but no details were given.  Cooking will neutralise potential microbial hazard while improving digestibility.  Cooking is also likely to reduce ANFs but excessive heat may promote Maillard and other reaction products that will reduce indispensable amino acid bioavailability. The METHODs should detail the legume variety, it should include a calculation of the digestibility-corrected amino acid score of the new formula and give an indication of the cooking/preparation procedure.

 Markers of improved body composition would seem an appropriate choice for primary outcome. Considering the likely resources in the field, MUAC seems entirely appropriate.  More accurate measures of Fat Free Mass gain are often too elaborate for use in a SAM setting.  Shorter term measures of ‘protein status’ may be caught within the biochemistry and/or metabolomics screens.

I have read this submission. I believe that I have an appropriate level of expertise to confirm that it is of an acceptable scientific standard.
